# A mark-specific quantile regression model

**DOI:** 10.1093/biomet/asad039

**Published:** 2023-06-20

**Authors:** Lianqiang Qu, Liuquan Sun, Yanqing Sun

**Affiliations:** School of Mathematics and Statistics, Central China Normal University, Wuhan, Hubei 430079, China; Institute of Applied Mathematics, Academy of Mathematics and Systems Science, Chinese Academy of Sciences, Beijing 100190, China; Department of Mathematics and Statistics, University of North Carolina at Charlotte, 9201 University City Boulevard, Charlotte, North Carolina 28223, USA

**Keywords:** Competing risk, Continuous mark, Hypothesis testing, Mark-specific quantile regression, Survival data, Vaccine efficacy

## Abstract

Quantile regression has become a widely used tool for analysing competing risk data. However, quantile regression for competing risk data with a continuous mark is still scarce. The mark variable is an extension of cause of failure in a classical competing risk model where cause of failure is replaced by a continuous mark only observed at uncensored failure times. An example of the continuous mark variable is the genetic distance that measures dissimilarity between the infecting virus and the virus contained in the vaccine construct. In this article, we propose a novel mark-specific quantile regression model. The proposed estimation method borrows strength from data in a neighbourhood of a mark and is based on an induced smoothed estimation equation, which is very different from the existing methods for competing risk data with discrete causes. The asymptotic properties of the resulting estimators are established across mark and quantile continuums. In addition, a mark-specific quantile-type vaccine efficacy is proposed and its statistical inference procedures are developed. Simulation studies are conducted to evaluate the finite sample performances of the proposed estimation and hypothesis testing procedures. An application to the first HIV vaccine efficacy trial is provided.

## 1 Introduction

### 1.1 Background

Quantile regression provides a comprehensive description of different parts of the conditional distribution of responses (Koenker & Bassett, 1978), and it has become a widely used tool in survival analysis. For example, Powell (1984, 1986) modified the least absolute deviation procedure to analyse censored observations. Portnoy (2003) developed a recursively reweighed estimation procedure by using the principle of self-consistency for the Kaplan–Meier estimator. Peng & Huang (2008) proposed a recursive series of estimating equations for a sequence of quantiles based on the martingale feature associated with censored data. De Backer et al. (2019) suggested an adaptive method to analyse survival data through modifying the so-called check function.

Competing risk data are common in survival analysis. When the competing causes of failures are finite, Peng & Fine (2007) proposed a nonparametric quantile inference method for cause-specific failure probabilities. Peng & Fine (2009) presented a competing risk quantile regression based on the cause-specific cumulative incidence function. Sun et al. (2012) developed a generalized linear quantile regression for competing risk data when the failure type may be missing. More related works are Lee & Han (2016), Ahn & Kim (2018), Choi et al. (2018) and Farcomeni & Geraci (2020), among others. Competing risk models with continuous causes of failure, or marks, are useful in many important applications (Sun et al., 2009, 2020). Our research is motivated by a dataset from an HIV vaccine efficacy trial, in which the vaccine may only provide protection for HIV strains genetically similar to the HIV virus or viruses represented in the vaccine. The similarity between the infecting virus and the virus contained in the vaccine construct can be measured by the genetic divergence, or distance. Thus, the genetic divergence of infecting HIV viruses from the HIV strain represented in the vaccine needs to be taken into account to properly assess vaccine efficacy.

**Fig. 1:**
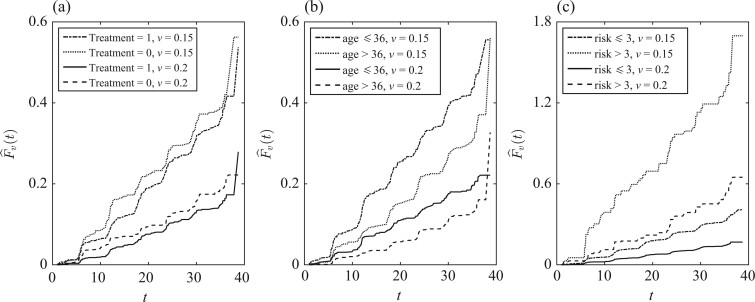
The estimated mark-specific cumulative incidence functions for the vaccine trial data. The mark-specific cumulative incidence function (Gilbert et al., 2008) is defined as $F_{v}(t)=\underset{h\to 0}{\lim}P(T\;⩽\;t,v\;⩽\;V<v+h)/h,$ where *T* is the time infected with HIV and *V* denotes the mark, i.e., weighted percent mismatch of amino acids.

The mark variable is a measure of the genetic distance between two aligned HIV sequences, which is defined as the weighted percent mismatch of amino acids between the two HIV sequences. Since this distance may be unique for all infected subjects and the genetic diversity of HIV is extensive, it is natural to consider the mark as a continuous variable. Furthermore, during the observing period, the volunteers are potentially at risk of HIV infection from more than one mutually exclusive strain of the virus, and the mark is only observed when HIV infection occurs. If HIV infection does not occur then the mark is undefined and is not meaningful. Thus, this situation can be considered a competing risk setting, where causes of failure are replaced by a continuous mark only observed at uncensored failure times, and the mark is considered as continuous causes of failure (Sun et al., 2009). A preliminary analysis of the data is given in Fig. 1, which plots the curves of the mark-specific cumulative incidence functions separately for subjects stratified by treatment, age at the median values and behavioural risk score (Gilbert et al., 2008). Figure 1 is quite suggestive of the effects of age and behavioural risk score on the mark-specific cumulative incidence functions.

When analysing continuous mark data, the existing methods developed for discrete competing risks can no longer be applied. First, the mark is from a continuous distribution, and observations at a specified value of the mark are sparse. This feature of data is very different from that of discrete competing risk data. In addition, inspecting Fig. 1 reveals that the effects of covariates on the conditional quantiles of the failure time may vary nonlinearly with the mark. But the methods of Peng & Fine (2009), Sun et al. (2012), Ahn & Kim (2018), Choi et al. (2018) and Farcomeni & Geraci (2020) assume that the effects of covariates are constant at each given quantile level. Therefore, suitable methods are needed to analyse the varying effects of covariates with the mark. Moreover, although marginal quantile regression methods such as in Portnoy (2003) and Peng & Huang (2008) can be used to assess the vaccine efficacy, it may fail to reveal the important relation between the vaccine efficacy and infecting viruses if the mark is ignored. A toy example is given near the end of § 1.3. The aim of this paper is to develop a quantile regression methodology for analysing survival data with continuous marks and assessing the HIV vaccine efficacy. The proposed method allows the covariate effects to vary nonlinearly with the mark.

### 1.2 Mark-specific quantile regression model

Let *T* be the failure time of interest and *C* be the censoring time. Let *V* denote a continuous mark variable and $Z={\left(1,{\tilde{Z}}^{T}\right)}^{T},$ where $\tilde{Z}$ is a *p*-dimensional covariate vector. Assume that *C* is independent of (*T*, *V*) given *Z*. Furthermore, denote by X=min(T,C) the observed time and by Δ=I(T ⩽ C) the censoring indicator, where I(·) denotes an indictor function. The mark is observed only when the corresponding failure time is uncensored. If Δ=0, then *V* is undefined and is not meaningful. The conditional mark-specific cumulative incidence function is defined as

$$F_{v}(t|Z)=\underset{h\to 0}{\lim}P(T\;⩽\;t,v\;⩽\;V<v+h|Z)/h.$$

The mark-specific cumulative incidence function is an extension of the cause-specific cumulative incidence function, where the cause of the failure time is replaced by a continuous mark (Gilbert et al., 2008). Suppose that $F_{v}(t|Z)\;⩽\;\tilde{\tau }$ for some constant $\tilde{\tau }>0,$ and that the support of mark *V* is taken to be [0,1], rescaling *V* if necessary. For v∈[0,1] and $\tau \in (0,\tilde{\tau }),$ we define the *τ*th conditional mark-specific quantile by

$$Q_{v}(\tau |Z)=\inf\{t\;F_{v}(t|Z)\;⩾\;\tau \}.$$

Under the competing risk framework, mark *V* is only meaningfully defined when failure occurs and it cannot be treated as a covariate. The proposed conditional mark-specific quantile $Q_{v}(\tau |Z)$ is not the conventional conditional quantile function $\tilde{Q}(\tau |Z,v)$ obtained by treating *V* as a covariate, where $\tilde{Q}(\tau |Z,v)=\inf\{t\;F(t|Z,v)\;⩾\;\tau \}$ and F(t|Z,v)=P(T ⩽ t|Z,V=v) is the conditional distribution of *T* given *Z* and V=v. The conditional distribution function F(t|Z,v), and therefore $\tilde{Q}(\tau |Z,v),$ is not identifiable under the competing risk setting.

The conditional mark-specific cumulative incidence function is an extension of the cause-specific cumulative incidence function in a competing risk setting, where the cause of the failure time is replaced by a continuous mark only observed at the failure time (Gilbert et al., 2008). Thus, the conditional mark-specific quantile function is an extension of the cause-specific quantile function in a competing risk setting for a continuous cause of failure, and is defined analogously to that for the competing risk data with finitely many competing risks (Peng & Fine, 2009). The conditional mark-specific quantile function $Q_{v}(\tau |Z)$ can be interpreted as the earliest time given covariate *Z* at which the proportion of subjects whose failures have occurred with mark *V* = *v* exceeds τ. For the HIV vaccine efficacy trials, $Q_{v}(\tau |Z)$ can be interpreted as the first time given covariate *Z* that the proportion of volunteers who have infected HIV with mark *V* = *v* exceeds τ.

Because $F_{v}(t|Z)=F(t|Z,v)f_{V}(v|Z),$ where $f_{V}(v|Z)$ is the conditional density function of *V* given *Z*, it follows that $Q_{v}(\tau |Z)=\tilde{Q}\{\tau^{*}(Z,v)|Z,v\}$ with $\tau^{*}(Z,v)=\tau /f_{V}(v|Z),$ and the range of *τ* is not necessarily bounded by 1. This is different from the competing risk quantile regression of Peng & Fine (2009) for the discrete mark in which case $f_{V}(v|Z)$ is the conditional probability mass function and $\tau =\tau^{*}(Z,v)f_{V}(v|Z)$ is bounded by 1 due to $0\;⩽\;\tau^{*}(Z,v)\;⩽\;1$. In what follows, we consider $\tau <\tilde{\tau }=\min_{v,z}\underset{t}{\max}F_{v}(t|Z=z)$; see § 2.3 for more details about the choice of the upper quantile.

We propose a novel mark-specified quantile regression model to capture the nonlinear interaction effects between the covariates and the mark on the failure. Specifically, for v∈[0,1] and $\tau \in (0,\tilde{\tau }),$ the model postulates that

(1) $$Q_{v}(\tau |Z)=\text{exp}\;\{Z^{T}\beta_{\tau }^{*}(v)\},$$

where $\beta_{\tau }^{*}(v)={\left\{\beta_{0\tau }^{*}(v),\beta_{1\tau }^{*}(v),\ldots ,\beta_{p\tau }^{*}(v)\right\}}^{T}$ is a (p+1)-dimensional vector of unknown continuous functions of *v* and τ, and characterizes the varying effects of *Z* on the conditional mark-specific quantile of the failure time with respect to *V*. By setting the first component of *Z* as 1, model (1) has a nonparametric baseline function $\text{exp}\;\{\beta_{0\tau }^{*}(v)\}$. Model (1) has a similar form to the varying-coefficient quantile regression model (Kim, 2007) in the absence of censored data:

$$\tilde{Q}(\tau |Z,v)=\text{exp}\;\{Z^{T}{\tilde{\beta }}_{\tau }^{*}(v)\}.$$

However, since $Q_{v}(\tau |Z)=\tilde{Q}\{\tau^{*}(Z,v)|Z,v\}$, $\tau^{*}(Z,v)=\tau /f_{V}(v|Z)$ depends on $f_{V}(v|Z)$ and (Z,v), and $f_{V}(v|Z)$ is not identifiable under the competing risk setting, the method of Kim (2007) cannot be directly applied to estimate ${\tilde{\beta }}_{\tau^{*}(Z,v)}^{*}(v)$ or $\beta_{\tau }^{*}(v)$ for model (1). In this paper, we develop an induced smoothing procedure (Brown & Wang, 2007) to estimate $\beta_{\tau }^{*}(v)$ under model (1), which is fast to implement using widely available numerical methods, such as the Newton–Raphson algorithm.

### 1.3 Quantile-type vaccine efficacy

The proposed model has applications in the sieve analysis of vaccine efficacies. To evaluate the HIV vaccine efficacy, write the covariate as $Z={\left(1,Z_{1},Z_{2}^{T}\right)}^{T},$ where *Z*_1_ is the treatment (vaccine) group indicator and *Z*_2_ is a vector of other covariates. We define the mark-specific quantile-type vaccine efficacy as

$$qve_{\tau }(v)=\frac{Q_{v}(\tau |1,Z_{1}=1,Z_{2})}{Q_{v}(\tau |1,Z_{1}=0,Z_{2})}-1.$$

Function $qve_{\tau }(v)$ characterizes the nonlinear dependence on *v* of the ratio of the conditional mark-specific quantile of the failure time at level *τ* under vaccine assignment $\left(Z_{1}=1\right)$ compared to under placebo assignment $(Z_{1}=0).$ A positive value of $qve_{\tau }(v)$ indicates that it takes a longer time to reach the same percentage (*τ*) of the mark-specific infections/diseases for the vaccine group as opposed to the placebo group. The larger the value of $qve_{\tau }(v)$, the more effective the vaccine. It is close to zero if and only if the conditional mark-specific quantiles of the failure time have no clear differences between the vaccine and placebo groups. Here we focus on the log-linear model for $Q_{v}(\tau |Z).$ This is because, under model (1), $qve_{\tau }(v)=\text{exp}\;\{\beta_{1\tau }^{*}(v)\}-1$ is free of *Z*_2_, but depends on the mark value *v*, which simplifies the inference procedure for $qve_{\tau }(v).$

The mark-specified quantile regression model (1) complements the modelling approaches based on the mark-specific hazard functions (Sun et al., 2009, 2020; Han et al., 2017) by allowing covariate effects varying over τ. It also complements the marginal quantile regression models (Portnoy, 2003; Peng & Huang, 2008) by providing additional insights on how the relation between the quantile and covariates changes with the mark. To illustrate the difference with the marginal quantile regression model, we consider a toy example. Let $\beta_{0\tau }^{*}(v)=\tau {\left(\gamma_{0}+v\right)}^{2}$ and $\beta_{1\tau }^{*}(v)=\tau \gamma_{1}\{2(\gamma_{0}+v)+\gamma_{1}\}.$ Assume that *V* is from *U*(0, 1), and that log(T) is from $U\{0,{\left(V+\gamma_{0}+\gamma_{1}Z_{1}\right)}^{2}\}$ given *V* and $Z_{1},$ where *U*(0, *c*) denotes a uniform distribution on (0,c),c>0. Thus, the marginal quantile of *T* given *Z*_1_ is $Q(\tau |Z_{1})=\text{exp}\;\{\tau \gamma_{0}(1+\gamma_{0})+\tau \gamma_{1}(1+2\gamma_{0}+\gamma_{1})Z_{1}\},$ where Q(τ|Z)=inf{t F(t|Z) ⩾ τ} and F(t|Z)=P(T ⩽ t|Z). If $\gamma_{1}=-(2\gamma_{0}+1)$ then $qve_{\tau }=Q(\tau |Z_{1}=1)/Q(\tau |Z_{1}=0)-1\equiv 0.$ But $qve_{\tau }(v)=\text{exp}\;\{\tau \gamma_{1}[2(\gamma_{0}+v)+\gamma_{1}]\}-1,$ which is not zero if $\gamma_{1}\neq 0.$ That is, the important vaccine effects can be missed without the consideration of the mark under this case.

We also consider a cumulative version of $qve_{\tau }(v),$ which is defined as $\mathrm{cqv}e_{\tau }(v)=\int_{a}^{v}qve_{\tau }(u)du$ with 0<a<1. The quantity can be used to assess the vaccine efficacy over a range of marks for v∈[a,b]⊂(0,1) and quantile levels $\tau \in [\tau_{0},\tau_{U}]$. We construct simultaneous confidence bands for $\mathrm{cqv}e_{\tau }(v),$ and propose test statistics to evaluate the mark-specific vaccine efficacy based on the estimator of $\mathrm{cqv}e_{\tau }(v)$ for v∈[a,b]⊂(0,1).

## 2 Estimation procedures

### 2.1 Induced smoothing estimators

Suppose that we observe *n* independent and identically distributed copies of (X,Δ,ΔV,Z), denoted by $(X_{i},\Delta_{i},\Delta_{i}V_{i},Z_{i}),i=1,\ldots ,n.$ In what follows, assume that the *T_i_* are continuous random variables. Let $N_{i}(t,v)=I(X_{i}\;⩽\;t,\Delta_{i}=1,V_{i}\;⩽\;v)$ be the marked point counting process with a jump at an uncensored failure time *X_i_* and the associated mark $V_{i}.$ Define $G(t|Z_{i})=P(C_{i}\;⩾\;t|Z_{i})$ as the survival function of the censoring time. Since *C_i_* is independent of (*T_i_*, *V _i_*) given $Z_{i},$ it can be checked that

$$F_{v}(t|Z_{i})=\underset{h\to 0}{\lim}\frac{1}{2h}E\{\int_{0}^{1}\int_{0}^{t}\frac{1}{G(s|Z_{i})}I(|u-v|\;⩽\;h)N_{i}(ds,du)|Z_{i}\},$$

which suggests that we can borrow strength from data in a neighbourhood of a mark. For each v∈(0,1), we propose the following mark-specific localized estimating equation to estimate $\beta_{\tau }^{*}(v)$:

$$U_{n}\{\beta_{\tau }(v)\}=0$$

with

$$U_{n}(\xi )=\frac{1}{n}\sum_{i=1}^{n}Z_{i}[\int_{0}^{1}\int_{0}^{L^{\;}}\frac{1}{G(t|Z_{i})}I\{\;\text{log}\;t\;⩽\;Z_{i}^{T}\xi \}K_{h}(u-v)N_{i}(dt,du)-\tau ].$$

Here $L^{\;}$ is the follow-up time satisfying $P(X_{i}\;⩾\;L)>0,\;K_{h}(x)=K(x/h)/h,\;K(x)$ is a kernel function with support on (−1,1) and *h* is a bandwidth.

Since $U_{n}(\xi )$ is monotone, but not continuous, an exact zero crossing of $U_{n}(\xi )$ may not exist. To be more specific, Fig. S1 in the Supplementary Material presents $U_{n}\{\beta_{\tau }(v)\}$ as a function of $\beta_{1\tau }^{*}(v)$ with τ=0.2 and *v* = 0.5 for model M3 studied in § 4, that is, $\beta_{0\tau }^{*}(v)$ and $\beta_{2\tau }^{*}(v)$ are assumed to be known. The sample size is 1500 and the bandwidth is 0.2. It can be seen that $U_{n}\{\beta_{\tau }(v)\}$ is very jagged and may flatten at 0.43, the value of $\beta_{1\tau }^{*}(0.5)$, which results in numerical challenges in computing the solution of $U_{n}(\xi ),$ particularly with multiple covariates. In addition, since $U_{n}(\xi )$ is nondifferentiable, the variance estimation of the resulting estimators can be very difficult.

To address these issues, we next propose an induced smoothing method to approximate $U_{n}(\xi )$ using continuously differentiable functions (Brown & Wang, 2007). Specifically, let $\Gamma_{\tau }(v)$ be a given (p+1)×(p+1) matrix such that $||\Gamma_{\tau }(v)|{|}_{F}=O(1),$ where $||D|{|}_{F}$ denotes the Frobenius norm of any matrix *D*. Similarly to Brown & Wang (2007), a smoothed version of $U_{n}\{\beta (v)\}$ can be constructed by $S_{n}\{\beta_{\tau }(v)\}=E_{W}[U_{n}\{\beta_{\tau }(v)+{\left(nh\right)}^{-1/2}\Gamma_{\tau }(v)W\}],$ where *W* is a random vector from $N(0,I_{p+1})$ independent of $(X_{i},\Delta_{i},\Delta_{i}V_{i},Z_{i}),$ and $I_{p+1}$ denotes the identity matrix of size p+1. A direct calculation shows that

$$S_{n}\{\beta_{\tau }(v)\}=\frac{1}{n}\sum_{i=1}^{n}Z_{i}[\int_{0}^{1}\int_{0}^{L^{\;}}\frac{1}{G(t|Z_{i})}\Phi \{\frac{Z_{i}^{T}\beta_{\tau }(v)-\text{log}\;t}{{\left(nh\right)}^{-1/2}\gamma_{i}}\}K_{h}(u-v)N_{i}(dt,du)-\tau ],$$

where Φ(x) denotes the cumulative function of the standard normal distribution and $\gamma_{i}=Z_{i}^{T}\Gamma_{\tau }(v)\Gamma_{\tau }{\left(v\right)}^{T}Z_{i}.$

In practice, the survival function G(t|Z) is usually unknown, but can be estimated from the observed data. For instance, if the censoring is dictated by administrative decisions or appears to be independent of the covariates, we can use the Kaplan–Meier estimator for the censoring distribution. When the censoring depends on the covariates, we can estimate G(t|Z) by specifying a semiparametric regression model for the censoring time, such as the Cox model. Here, for notational simplicity, we just consider the censoring to be independent of the covariates, but the estimation procedures are similar for the case when the censoring depends on the covariates. Let *G*(*t*) be the survival function of the censoring time; we focus on the Kaplan–Meier estimator of G(t), denoted $\hat{G}(t).$ In addition, in the numerical studies below, we take $\Gamma_{\tau }(v)=I_{p+1}$ for computational convenience.

Let ${\hat{S}}_{n}(b)$ denote the estimating equation by replacing $G(t|Z_{i})$ with $\hat{G}(t)$ in $S_{n}(b).$ We can show that ${\hat{S}}_{n}\{\beta_{\tau }(v)\}$ is generally close to $U_{n}\{\beta_{\tau }(v)\}.$ This fact can also be confirmed in the aforementioned Fig. S1 in the Supplementary Material, where ${\hat{S}}_{n}\{\beta_{\tau }(v)\}$ approximates $U_{n}\{\beta_{\tau }(v)\}$ well and has a unique solution. In what follows, we propose to estimate $\beta_{\tau }^{*}(v)$ by the solution to ${\hat{S}}_{n}\{\beta_{\tau }(v)\}=0,$ denoted ${\hat{\beta }}_{\tau }(v).$

### 2.2 Asymptotic properties

We establish the uniform consistency and asymptotic normality of ${\hat{\beta }}_{\tau }(v)$ for $(v,\tau )\in B=[a,b]\times [\tau_{0},\tau_{U}],$ where 0<a<b<1 and $0<\tau_{0}<\tau_{U}<\tilde{\tau }.$ We assume that the following conditions hold.

Condition 1. We have P(X ⩾ L)>0, and *Z* is bounded almost surely.

Condition 2. Each component of $\beta_{\tau }^{*}(v)$ is Lipschitz continuous with respect to (v,τ)∈B.

Condition 3. The conditional density function f(t,v|z) of (*T*, *V*) given *Z* = *z* is twice continuously differentiable with respect to *t* and *v*, and is bounded uniformly in (t,v,z)∈[0,L]×[a,b]×C, where C denotes the support of *Z*. In addition, the conditional density function g(v|z,t) of *V* given (Z,T)=(z,t) is bounded away from 0 and ∞ uniformly in (t,v,z)∈[0,L]×[a,b]×C.

Condition 4. It holds that $\underset{(v,\tau )\in B}{\inf}\lambda_{\text{min}}\{A_{\tau }(v)\}>0,$ where $\lambda_{\text{min}}(\cdot )$ denotes the minimum eigenvalue of a matrix, and

$$A_{\tau }(v)=E(ZZ^{T}\;\text{exp}\;\{Z^{T}\beta_{\tau }^{*}(v)\}f[\text{exp}\;\{Z^{T}\beta_{\tau }^{*}(v)\},v|Z]).$$

Condition 5. The kernel function *K*(*x*) is symmetric with support [−1,1], and has bounded variation satisfying ∫K(u)du=1.

Condition 6. The bandwidth satisfies $nh^{2}\to \infty$ and $nh^{5}\to 0.$

Conditions 1–4 are standard assumptions for quantile regression methods in the context of survival analysis, which are analogous to those in Peng & Huang (2008) and Qian & Peng (2010). Conditions 2 and 3 are needed for the uniform consistency and the asymptotic normality of ${\hat{\beta }}_{\tau }(v).$ Condition 4 ensures the identifiability of $\beta_{\tau }^{*}(v).$ Conditions 5 and 6 are standard assumptions for kernel smoothing techniques. The uniform consistency and asymptotic normality of ${\hat{\beta }}_{\tau }(v)$ are given in the following two theorems.

THEOREM 1. *Suppose that Conditions 1–6 hold. Then* $\left||{\hat{\beta }}_{\tau }(v)-\beta_{\tau }^{*}(v)||=o_{p}(1\right)$*uniformly in* (v,τ)∈B.

THEOREM 2. *Suppose that Conditions 1–6 hold. Then* ${\left(nh\right)}^{1/2}\{{\hat{\beta }}_{\tau }(v)-\beta_{\tau }^{*}(v)\}$*converges in distribution to a zero-mean normal random vector with covariance matrix* $\Omega_{\tau }(v)$*for* (v,τ)∈B,*where* $\Omega_{\tau }(v)=A_{\tau }{\left(v\right)}^{-1}D(v,\tau )A_{\tau }{\left(v\right)}^{-1},$

$$D(v,\tau )=\nu_{0}E[ZZ^{T}I\{\;\text{log}\;T\;⩽\;Z^{T}\beta_{\tau }^{*}(v)\}G{\left(T\right)}^{-1}g(v|Z,T)]$$

*and* $\nu_{0}=\int K{\left(u\right)}^{2}du.$

In order to estimate $\Omega_{\tau }(v),$ we need to estimate $A_{\tau }(v)$ and $D_{\tau }(v,\tau ).$ First, by checking the proofs of Theorems 1 and 2, we can consistently estimate $A_{\tau }(v)$ by

$${\hat{A}}_{\tau }(v)=n^{-1/2}h^{1/2}\sum_{i=1}^{n}\int_{0}^{1}\int_{0}^{L^{\;}}\frac{Z_{i}Z_{i}^{T}}{\hat{G}(t)\gamma_{i}}\phi \{\frac{Z_{i}^{T}{\hat{\beta }}_{\tau }(v)-\text{log}\;t}{{\left(nh\right)}^{-1/2}\gamma_{i}}\}K_{h}(u-v)N_{i}(dt,du),$$

where ϕ(x) is the density function of the standard normal distribution. In addition, we can consistently estimate D(v,τ) by

$$\hat{D}(v,\tau )=n^{-1}h\sum_{i=1}^{n}{\hat{\eta }}_{i}(v,\tau ){\hat{\eta }}_{i}{\left(v,\tau \right)}^{T},$$

where

$${\hat{\eta }}_{i}(v,\tau )=Z_{i}[\int_{0}^{1}\int_{0}^{L}I\{\;\text{log}\;t\;⩽\;Z_{i}^{T}{\hat{\beta }}_{\tau }(v)\}K_{h}(u-v)\hat{G}{\left(t\right)}^{-1}N_{i}(dt,du)-\tau ].$$

Thus, $\Omega_{\tau }(v)$ can be consistently estimated by ${\hat{\Omega }}_{\tau }(v),$ where

(2) $${\hat{\Omega }}_{\tau }(v)={\hat{A}}_{\tau }{\left(v\right)}^{-1}\hat{D}(v,\tau ){\hat{A}}_{\tau }{\left(v\right)}^{-1}.$$

Remark 1. Theorem 2 implies that the covariance matrix of ${\hat{\beta }}_{\tau }(v)$ is not affected asymptotically by $\hat{G}(t).$ One intuitive explanation is that $\hat{G}(t)$ is irrelevant to the mark in estimating ${\hat{\beta }}_{\tau }(v),$ and the former converges at a faster rate. Similar results have also been obtained by Zhang et al. (2022) for quantile regression models, where the inference on the parameters of interest is not affected asymptotically by the estimation of nuisance parameters.

Remark 2. As suggested by Brown & Wang (2007), we can also apply an iterative procedure to solve ${\hat{S}}_{n}\{\beta_{\tau }(v)\}=0,$ which can simultaneously estimate $\beta_{\tau }^{*}(v)$ and the asymptotic covariance matrix. Specifically, define $\beta_{\tau }^{\left(k\right)}(v)$ as the estimate of $\beta_{\tau }^{*}(v)$ at the *k*th iteration.

*Step* 0. “Choose an initial estimate $\beta_{\tau }^{\left(0\right)}(v)$ by using the solution to ${\hat{S}}_{n}\{\beta_{\tau }(v)\}=0$ with $\Gamma_{\tau }(v)=I_{p+1},$ and let k=0.

*Step* 1.Update $\Gamma_{\tau }(v)={\left[{\hat{\Omega }}_{\tau }^{\left(k\right)}(v)\right]}^{1/2},$ where ${\hat{\Omega }}_{\tau }^{\left(k\right)}(v)$ is obtained by replacing ${\hat{\beta }}_{\tau }(v)$ with $\beta_{\tau }^{\left(k\right)}(v)$ in ${\hat{\Omega }}_{\tau }(v)$ defined by (2)

*Step* 2.Obtain $\beta_{\tau }^{\left(k+1\right)}(v)$ by solving ${\hat{S}}_{n}\{\beta_{\tau }(v)\}=0$ with $\Gamma_{\tau }(v)={\left[{\hat{\Omega }}_{\tau }^{\left(k\right)}(v)\right]}^{1/2}.$

*Step* 3.Set k=k+1. Repeat steps 1 and 2 until convergence of both $\beta_{\tau }^{\left(k\right)}(v)$ and ${\hat{\Omega }}_{\tau }^{\left(k\right)}(v)$ is achieved to a specified tolerance.

However, this procedure is very time consuming for estimating $\beta_{\tau }^{*}(v)$ with respect to *v* and *τ*.

Remark 3. Our proposed method can be extended to other link functions for $Q_{v}(\tau |Z).$ A more general approach is to generalize model (1) to

$$Q_{v}(\tau |Z)=H\{Z^{T}\beta_{\tau }^{*}(v)\},$$

where H(·)>0 is a known monotone link function (Peng & Fine, 2009; Sun et al., 2012). Under this setting, the estimating equation takes the form

$$\frac{1}{n}\sum_{i=1}^{n}Z_{i}[\int_{0}^{1}\int_{0}^{L^{\;}}\frac{1}{\hat{G}(t)}\Phi \{\frac{Z_{i}^{T}\beta_{\tau }(v)-H^{-1}(t)}{{\left(nh\right)}^{-1/2}\gamma_{i}}\}K_{h}(u-v)N_{i}(dt,du)-\tau ]=0.$$

Let $A_{\tau }^{*}(v)=E(ZZ^{T}\dot{H}\{Z^{T}\beta_{\tau }^{*}(v)\}f[H\{Z^{T}\beta_{\tau }^{*}(v)\},v|Z])$ and

$$D^{*}(v,\tau )=\nu_{0}E[ZZ^{T}I\{H^{-1}(T)\;⩽\;Z^{T}\beta_{\tau }^{*}(v)\}G{\left(T\right)}^{-1}g(v|Z,T)],$$

where $\dot{H}(x)$ denotes the first derivative of H(x). Then Theorems 1 and 2 still hold by replacing $A_{\tau }(v)$ and D(v,τ) with $A_{\tau }^{*}(v)$ and $D^{*}(v,\tau ),$ respectively.

Let $B_{\tau }^{*}(v)=\int_{a}^{v}\beta_{\tau }^{*}(u)du$ denote the cumulative regression coefficient, which can be estimated by ${\hat{B}}_{\tau }(v)=\int_{a}^{v}{\hat{\beta }}_{\tau }(u)du.$ Define $\omega_{\tau }(s,u)=E[ZI[s\;⩽\;T\;⩽\;\;\text{exp}\;\{Z^{T}\beta_{\tau }^{*}(u)\}]g(u|Z,T)],\;y(t)=P(X\;⩾\;t)$ and $M^{C}(t)=N^{C}(t)-\int_{0}^{t}Y(s)d\Lambda^{C}(s),$ where $N^{C}(t)=I(X\;⩽\;t,\Delta =0),\;Y(t)=I(X\;⩾\;t)$ and $\Lambda^{C}(t)$ is the cumulative hazard function of the censoring time. The following theorem establishes the weak convergence of ${\hat{B}}_{\tau }(v).$

THEOREM 3. *Suppose that Conditions 1–6 hold. Then* $n^{1/2}\{{\hat{B}}_{\tau }(v)-B_{\tau }^{*}(v)\}$*converges weakly on* $\left[a,b]\times [\tau_{0},\tau_{U}\right]$*to a* (p+1)*-dimensional Gaussian process with mean zero and covariance matrix* $\Psi (v_{1},v_{2},\tau_{1},\tau_{2})=E\{\varphi_{1}(v_{1},\tau_{1})\varphi_{1}{\left(v_{2},\tau_{2}\right)}^{T}+\varphi_{2}(v_{1},\tau_{1})\varphi_{2}{\left(v_{2},\tau_{2}\right)}^{T}\}$*at* $\left(v_{1},\tau_{1}\right)$*and* $(v_{2},\tau_{2}),$*where*

$$\begin{array}{c} \varphi_{1}(v,\tau )=[\int_{a}^{v}\int_{0}^{L}A_{\tau }{\left(u\right)}^{-1}I\{\;\text{log}\;t\;⩽\;Z^{T}\beta_{\tau }^{*}(u)\}\frac{N(dt,du)}{G(t)}-\tau \int_{a}^{v}A_{\tau }{\left(u\right)}^{-1}du]Z,\\ \varphi_{2}(v,\tau )=\int_{a}^{v}\int_{0}^{L}A_{\tau }{\left(u\right)}^{-1}\omega_{\tau }(s,u)y{\left(s\right)}^{-1}dM^{C}(s)du, \end{array}$$

*and* $\varphi_{1}(v,\tau )$*and* $\varphi_{2}(v,\tau )$*are independent of each other.*

The covariance function $\Psi (v_{1},v_{2},\tau_{1},\tau_{2})$ can be consistently estimated by

$$\hat{\Psi }(v_{1},v_{2},\tau_{1},\tau_{2})=\frac{1}{n}\sum_{i=1}^{n}[{\hat{\varphi }}_{1i}(v_{1},\tau_{1}){\hat{\varphi }}_{1i}{\left(v_{2},\tau_{2}\right)}^{T}+{\hat{\varphi }}_{2i}(v_{1},\tau_{1}){\hat{\varphi }}_{2i}{\left(v_{2},\tau_{2}\right)}^{T}],$$

where

$$\begin{array}{c} {\hat{\varphi }}_{1i}(v,\tau )=[\int_{a}^{v}\int_{0}^{L}{\hat{A}}_{\tau }{\left(u\right)}^{-1}I\{\;\text{log}\;t\;⩽\;Z_{i}^{T}{\hat{\beta }}_{\tau }(u)\}\frac{N_{i}(dt,du)}{\hat{G}(t)}-\tau \int_{a}^{v}{\hat{A}}_{\tau }{\left(u\right)}^{-1}du]Z_{i},\\ {\hat{\varphi }}_{2i}(v,\tau )=\int_{a}^{v}\int_{0}^{L}{\hat{A}}_{\tau }{\left(u\right)}^{-1}{\hat{\omega }}_{\tau }(s,u)\bar{Y}{\left(s\right)}^{-1}d{\hat{M}}_{i}^{C}(s)du,\\ {\hat{\omega }}_{\tau }(s,v)=\frac{1}{n}\sum_{i=1}^{n}\int_{a}^{b}\int_{0}^{L}[Z_{i}I[s\;⩽\;t\;⩽\;\;\text{exp}\;\{Z_{i}^{T}{\hat{\beta }}_{\tau }(v)\}]K_{h}(u-v)\frac{N_{i}(dt,du)}{\hat{G}(t)}], \end{array}$$

$\bar{Y}(s)=n^{-1}\sum_{i=1}^{n}I(X_{i}\;⩾\;s),\;{\hat{M}}_{i}^{C}(s)=I(X_{i}\;⩽\;s,\Delta_{i}=0)-\int_{0}^{s}Y_{i}(u)d{\hat{\Lambda }}^{C}(u)$ and ${\hat{\Lambda }}^{C}(s)$ is the Nelson–Aalen estimator of $\Lambda^{C}(s).$

### 2.3 Tuning parameter selection

We provide some practical guidance on how to select the tuning parameters, including the bandwidth parameter used in induced smoothing, the range of quantile levels and the range of *V*. Bandwidth selection is often a critical part of nonparametric regression. Here we use an *M*-fold cross-validation method to choose the bandwidth parameter (Tian et al., 2005). Specifically, we randomly divide the data into *M* roughly equal-sized groups. Let *D_k_* denote the *k*th subgroup of data and ${\hat{\beta }}_{\tau }^{\left(-k\right)}(v)$ be an estimate of $\beta_{\tau }^{*}(v)$ using the data from all subgroups other than $D_{k}.$ Under model (1), we have

$$E[\int_{0}^{v}\int_{0}^{\;\text{exp}\;\{Z_{i}^{T}\beta_{\tau }^{*}(u)\}}\frac{N_{i}(ds,du)}{G(s|Z_{i})}|Z_{i}]=\int_{0}^{v}F_{u}[\text{exp}\;\{Z_{i}^{T}\beta_{\tau }^{*}(u)\}|Z_{i}]du=\tau v.$$

Thus, the *k*th prediction error is given by

$${\text{PE}}_{k}(h)=\sum_{i\in D_{k}}\int_{\tau_{0}}^{\tau_{U}}\int_{a}^{b}[\int_{0}^{v}\int_{0}^{L}\frac{I\{\;\text{log}\;s\;⩽\;Z_{i}^{T}{\hat{\beta }}_{\tau }^{\left(-k\right)}(u)\}}{\hat{G}(s)}N_{i}(ds,du)-\tau v{]}^{2}dvd\tau .$$

The optimal bandwidth is obtained as

$$h_{\text{opt}}=\text{arg}{\text{min}}_{h}\sum_{k=1}^{M}{\text{PE}}_{k}(h).$$

In practice, however, the cross-validation method may be time consuming. Alternatively, we could choose the bandwidth $h=\varpi {\hat{\sigma }}_{V}n_{0}^{-1/4}$ by using the rule-of-thumb bandwidth, where ${\hat{\sigma }}_{V}$ is the estimated standard error of the observed marks, *n*_0_ is the number of observed failure times and ϖ>0 is a prespecified constant (Sun et al., 2009; Han et al., 2017). Simulation results presented in § 4 show that the bandwidth $h_{\text{opt}}$ leads to smaller estimated standard deviation in estimation and higher power for the tests.

For the range of quantile levels, the lower and upper quantiles, *τ*_0_ and *τ_U_*, are required to satisfy the conditions that there is no censoring below the *τ*_0_th quantile and $\tau_{U}<\min_{v,z}\max_{t}F_{v}(t|Z=z).$ In practice, *τ*_0_ can be chosen to be close to 0 if censoring occurs at early stages (He et al., 2022). The upper quantile *τ_U_* can be chosen such that $\tau_{U}<\min_{v,z}\max_{t}{\hat{F}}_{v}(t|Z=z),$ where ${\hat{F}}_{v}(t|Z=z)$ is an estimate of $F_{v}(t|Z=z).$ For a discrete covariate *Z* or a stratified version based on $Z,\;F_{v}(t|Z=z)$ can be estimated by

$${\hat{F}}_{v}(t|Z=z)=\frac{1}{n}\sum_{i=1}^{n}\int_{0}^{1}\int_{0}^{t}\frac{I(Z_{i}=z)}{\hat{G}(s)}K_{h}(u-v)N_{i}(ds,du).$$

By plotting the mark-specific cumulative incidence function estimates stratified on covariates as in Fig. 1, for example, we can choose *τ_U_* such that all estimated curves exceed it in the right tails.

For the range of *V*, as discussed in Sun et al. (2009), we assume that the mark variable *V* has a known and bounded support, and that, without loss of generality, this support is taken to be [0,1], rescaling *V* if necessary (Sun et al., 2009). In practice, the range of *V* can be taken to be $\left(V_{\text{min}},V_{\text{max}}\right)$ and $[a,b]\subset (V_{\text{min}},V_{\text{max}}),$ where $V_{\text{min}}=\min_{i\;\delta_{i}=1}V_{i}$ and $V_{\text{max}}=\max_{i\;\delta_{i}=1}V_{i}.$

## 3 Inference for vaccine efficacy

The confidence bands for the regression coefficients and the mark-specific quantile-type vaccine efficacy are provided in the Supplementary Material. Here we test the mark-specific quantile-type vaccine efficacy. If the vaccine has no efficacy then the vaccine will provide no protection against any infecting strain of the virus. As a result, the *τ*th mark-specific quantiles should have no significant difference between the vaccine and placebo groups for all v∈[a,b] and $\tau \in [\tau_{0},\tau_{U}]$. That is, $qve_{\tau }(v)\equiv 0$ for all (v,τ)∈B. Thus, it is of interest to test the efficacy over a range of *v* and *τ* to assess the overall clinical/public health benefit of the vaccine. The first set of hypotheses is

$$\begin{array}{c} H_{10}\;qve_{\tau }(v)\equiv 0\;\text{for all}\;(v,\tau )\in B\\ \text{versus}\;H_{11}\;qve_{\tau }(v)\neq 0\;\text{for some}\;(v,\tau )\in B\\ \text{or}\;H_{12}:\;\text{for each given}\;\tau ,qve_{\tau }(v)\;⩾\;0\;\text{with strict inequality for at least some}\;v. \end{array}$$

To test $H_{10},$ we consider the statistics

$$T_{11}=\underset{\tau \in [\tau_{0},\tau_{U}]}{\sup}\int_{a}^{b}\frac{n{\left\{{\hat{\mathrm{cqve}}}_{\tau }(v)\right\}}^{2}}{{\hat{\zeta }}_{0\tau }(v)}dv\;\text{and}\;T_{12}(\tau )=\frac{n^{1/2}{\hat{\mathrm{cqve}}}_{\tau }(b)}{{\hat{\zeta }}_{0\tau }^{1/2}(b)},$$

where ${\hat{\zeta }}_{0\tau }(v)=n^{-1}\sum_{i=1}^{n}{\hat{\vartheta }}_{0i}^{2}(\tau ,v)$ with

(3) $${\hat{\vartheta }}_{0i}(\tau ,v)=-\int_{a}^{v}\;\text{exp}\;\{{\hat{\beta }}_{1\tau }(u)\}e_{1}^{T}{\hat{A}}_{\tau }{\left(u\right)}^{-1}[{\hat{\varphi }}_{1i}(v,\tau )+{\hat{\varphi }}_{2i}(v,\tau )]du$$

and $e_{1}={\left(0,1,0,\ldots ,0\right)}^{T}\in R^{p+1}.$ The test statistic $T_{11}$ captures general departures $H_{11},$ while $T_{12}(\tau )$ is sensitive to the alternative $H_{12},$ which is likely to be positive when *H*_12_ holds. The statistics $T_{11}$ and $T_{12}$ are close to zero when $qve_{\tau }(v)\equiv 0,$ and hence we reject *H*_10_ if $T_{11}>c_{11}(\alpha )$ and $T_{12}(\tau )>c_{12}(\alpha ),$ where $c_{11}(\alpha )$ and $c_{12}(\alpha )$ are the critical values. By some arguments similar to those of § 2.2, under *H*_10_, $n^{1/2}{\hat{\mathrm{cqve}}}_{\tau }(v)$ is asymptotically equivalent to $n^{-1/2}\sum_{i=1}^{n}{\hat{\vartheta }}_{0i}(\tau ,v),$ where ${\hat{\vartheta }}_{0i}(\tau ,v)$ is defined in (3). Thus, $c_{12}(\alpha )$ can be taken as the upper *α*-quantile $z_{\alpha }$ of the standard normal distribution. To obtain the critical value $c_{11}(\alpha ),$ we consider a resampling technique (Lin et al., 1993). Let

$$T_{11}^{*}=\underset{\tau \in [\tau_{0},\tau_{U}]}{\sup}\int_{a}^{b}\frac{{\left\{n^{-1/2}\sum_{i=1}^{n}W_{i}{\hat{\vartheta }}_{0i}(\tau ,v)\right\}}^{2}}{{\hat{\zeta }}_{0\tau }(v)}dv,$$

where $W_{i},i=1,\ldots ,n,$ are independent standard normal variables and are independent of the observed data. According to the arguments of Lin et al. (1993), the null distribution of $T_{11}$ can be approximated by the conditional distribution of $T_{11}^{*}$ given the observed data, which can be obtained by repeatedly generating the normal random sample $W_{i},i=1,\ldots ,n$, while fixing the observed data. Thus, the critical values $c_{11}(\alpha )$ can be taken as the (1−α)-percentile of the conditional distributions of $T_{11}^{*}.$

When the null *H*_10_ is rejected, one may wish to test whether the quantile-type vaccine efficacy varies with respect to *v* and τ. In addition, if the vaccine is effective then the vaccine will afford protection against the infecting strain of the virus, and the vaccine efficacy will decrease as *v* increases (Sun et al., 2009). This leads to $qve_{\tau }(v)$ decreasing with *v* for each given τ. Thus, we consider the following set of hypotheses:

$$\begin{array}{c} H_{20}\;qve_{\tau }(v)=\psi \;\text{for all}\;(v,\tau )\in B\\ \text{versus}\;H_{21}\;qve_{\tau }(v)\neq \psi \;\text{for some}\;(v,\tau )\in B\\ \text{or}\;H_{22}\;\text{for each given}\;\tau ,qve_{\tau }(v)\;\text{decreases as}\;v\;\text{increases} \end{array}$$

with *ψ* some unspecified constant.

Let *a*_1_ and $\tau_{0}^{*}$ be two specified constants such that $a<a_{1}<b$ and $\tau_{0}<\tau_{0}^{*}<\tau_{U}.$ To test $H_{20},$ we propose the test statistics

$$T_{21}(\tau ,v)=n^{1/2}\{\frac{{\hat{\mathrm{ccqve}}}_{\tau }(v)}{\left(v-a)(\tau -\tau_{0}\right)}-\frac{{\hat{\mathrm{ccqve}}}_{\tau_{U}}(b)}{\left(b-a)(\tau_{U}-\tau_{0}\right)}\}/{\hat{\zeta }}_{1\tau }^{1/2}(v)$$

and

$$T_{22}(\tau )=n^{1/2}\int_{a_{1}}^{b}\{(\frac{{\hat{\mathrm{cqve}}}_{\tau }(v)}{v-a}-\frac{{\hat{\mathrm{cqve}}}_{\tau }(b)}{b-a})/{\hat{\zeta }}_{2\tau }^{1/2}(v)\}dv,$$

where ${\hat{\mathrm{ccqve}}}_{\tau }(v)=\int_{\tau_{0}}^{\tau }{\hat{\mathrm{cqve}}}_{s}(v)ds$, ${\hat{\zeta }}_{1\tau }(v)=n^{-1}\sum_{i=1}^{n}{\hat{\vartheta }}_{1i}^{2}(\tau ,v)$ and ${\hat{\zeta }}_{2\tau }(v)=n^{-1}\sum_{i=1}^{n}{\hat{\vartheta }}_{2i}^{2}(\tau ).$ Here, ${\hat{\vartheta }}_{1i}(\tau ,v)$ and ${\hat{\vartheta }}_{2i}(\tau )$ are defined as

$${\hat{\vartheta }}_{1i}(\tau ,v)=\frac{\int_{\tau_{0}}^{\tau }{\hat{\vartheta }}_{0i}(s,v)ds}{\left(v-a)(\tau -\tau_{0}\right)}-\frac{\int_{\tau_{0}}^{\tau_{U}}{\hat{\vartheta }}_{0i}(s,b)ds}{\left(b-a)(\tau_{U}-\tau_{0}\right)}$$

and

$${\hat{\vartheta }}_{2i}(\tau )=\int_{a_{1}}^{b}\{\frac{{\hat{\vartheta }}_{0i}(\tau ,v)}{v-a}-\frac{{\hat{\vartheta }}_{0i}(\tau_{U},b)}{b-a}\}dv.$$

We further define

$$T_{21}=\underset{v\in [a_{1},b]}{\sup}\underset{\tau \in [\tau_{0}^{*},\tau_{U}]}{\sup}|T_{21}(\tau ,v)|.$$

When $qve_{\tau }(v)$ is a constant function, $T_{21}$ and $T_{22}(\tau )$ are likely to be zero. The statistic $T_{21}$ captures general departure $H_{21},$ while $T_{22}(\tau )$ is sensitive to the monotone alternative $H_{22},$ which is likely to be positive when *H*_22_ holds. Hence, we reject *H*_20_ if $T_{21}>c_{21}(\alpha )$ and $T_{22}(\tau )>c_{22}(\alpha ),$ where $c_{21}(\alpha )$ and $c_{22}(\alpha )$ are the critical values. Under $H_{20},$$T_{22}(\tau )$ is asymptotically standard normal, and $c_{22}(\alpha )$ can be taken as the upper *α*-quantile $z_{\alpha }$ of the standard normal distribution. The critical value $c_{21}(\alpha )$ can be obtained through the following resampling technique. Let

$$T_{21}^{*}=\underset{v\in [a_{1},b]}{\sup}\underset{\tau \in [\tau_{0}^{*},\tau_{U}]}{\sup}|n^{-1/2}\sum_{i=1}^{n}W_{i}{\hat{\vartheta }}_{1i}(\tau ,v)/{\hat{\zeta }}_{1\tau }^{1/2}(v)|.$$

Then the null distribution of $T_{21}$ can be approximated by the conditional distribution of $T_{21}^{*}$ given the observed data, and the critical value $c_{21}(\alpha )$ can be taken as the (1−α)-percentile of the conditional distribution of $T_{21}^{*}.$

## 4 Simulation studies

In this section, we conduct simulation studies to evaluate the finite sample performance of the proposed method. We first generate $Z^{*}=(Z_{1}^{*},Z_{2}^{*})$ from a multivariate normal distribution with mean 0, variance 1 and correlation 0.5. Then let ${\tilde{Z}}_{1}=I(Z_{1}^{*}>0)$ and ${\tilde{Z}}_{2}=\Phi (Z_{2}^{*}).$ Under these settings, ${\tilde{Z}}_{1}$ follows a Bernoulli distribution with success probability $0.5,\;{\tilde{Z}}_{2}$ follows a uniform distribution *U*(0, 1) and ${\tilde{Z}}_{1}$ is related to ${\tilde{Z}}_{2}.$ The conditional density of *V* equals 1 if $\mu {\tilde{Z}}_{1}=0$ and is 2*v* otherwise, where *μ* is a constant given below. The failure time *T* satisfies $P(T\;⩽\;t|V,\tilde{Z})=\Phi \{\;\text{log}\;t-\gamma {\left(V\right)}^{T}\tilde{Z}\},$ where $\gamma (v)={\left\{\gamma_{1}(v),\gamma_{2}(v)\right\}}^{T}.$ Let $Z={\left(1,{\tilde{Z}}^{T}\right)}^{T}$ with $\tilde{Z}={\left({\tilde{Z}}_{1},{\tilde{Z}}_{2}\right)}^{T}$. Under the preceding settings, the underlying quantile regression model takes the form

$$\text{log}\;\{Q_{v}(\tau |Z)\}=\Phi^{-1}(\tau )+[\gamma_{1}(v)+\mu \{\Phi^{-1}(\frac{\tau }{2v})-\Phi^{-1}(\tau )\}]{\tilde{Z}}_{1}+\gamma_{2}(v){\tilde{Z}}_{2}.$$

Here, we set $\gamma_{1}(v)=\gamma_{11}+\gamma_{12}v$ and $\gamma_{2}(v)=0.5(1+v^{2}).$ In the study, four models are considered:

M1: $\left(\mu ,\gamma_{11},\gamma_{12})=(0,0,0\right)$,

M2: $\left(\mu ,\gamma_{11},\gamma_{12})=(0,0.4,0\right)$,

M3: $\left(\mu ,\gamma_{11},\gamma_{12})=(1,0.43,0\right)$,

M4: $\left(\mu ,\gamma_{11},\gamma_{12})=(1,0.9,-0.6\right)$.

Model M1 is considered for the null hypothesis *H*_10_ of no vaccine efficacy, and models M2–M4 are considered for the alternative hypotheses *H*_11_ and $H_{12}.$ The departure from *H*_10_ increases as the model moves from M2 to M4. Model M2 is also considered for the null hypothesis *H*_20_ of constant vaccine efficacy, while M3–M4 are considered for the alternatives *H*_21_ and $H_{22}.$ The departure from *H*_20_ increases as the model moves from M3 to M4. The censoring time *C* is generated from an exponential distribution with mean *c*, where *c* is chosen to give a censoring rate of about 40% under models M1–M4.

Set [a,b]=[0.3,0.8] and $[\tau_{0},\tau_{U}]=[0.1,0.4].$ For the calculations of ${\hat{\mathrm{cqve}}}_{\tau }(v)$ and the tests studied in § 3, we take a grid of 50 evenly spaced points in [a,b] and $[\tau_{0},\tau_{U}].$ In the test statistics $T_{21}$ and $T_{22}(\tau ),$ the parameters *a*_1_ and $\tau_{0}^{*}$ are taken as 0.35 and 0.15, respectively. The kernel function is set to be the Epanechnikov kernel function, that is, $K(x)=0.75(1-x^{2})I(|x|<1).$ The bandwidth *h* is chosen using the five-fold cross-validation method, and the optimal bandwidth is denoted by $h_{\text{opt}}.$ For a sensitivity analysis, we also consider *h* = 0.15 or 0.2 by using the rule-of-thumb bandwidth. The critical values are calculated using the resampling method with 1000 simulated realizations. The results presented below are based on 1000 replications with sample sizes *n* = 1000 and 1500.

Table 1 and the tables in the Supplementary Material report the empirical biases, the empirical standard deviations, the average of the estimated standard deviations of ${\hat{\mathrm{qve}}}_{\tau }(v)$ and ${\hat{\mathrm{cqve}}}_{\tau }(v),$ and the coverage probabilities of the pointwise 95% confidence bands for $qve_{\tau }(v)$ and $\mathrm{cqv}e_{\tau }(v).$ The results suggest that the proposed estimators perform reasonably well. Specifically, the proposed estimators are nearly unbiased, the estimated standard deviations agree well with the empirical standard deviations and the coverage probabilities of the pointwise 95% confidence bands are close to the nominal level. The performance of the proposed estimators becomes better when the sample size increases from 1000 to 1500. In addition, the optimal bandwidth $h_{\text{opt}}$ leads to smaller estimated standard deviations for ${\hat{\mathrm{cqve}}}_{\tau }(v)$ and ${\hat{\mathrm{qve}}}_{\tau }(v)$ than *h* = 0.15 and 0.2.

**Table 1:** Simulation results for ${\hat{\mathrm{qve}}}_{\tau }(v)$ and ${\hat{\mathrm{cqve}}}_{\tau }(v)$ under model M1

				${\hat{\mathrm{qve}}}_{\tau }(v)$				${\hat{\mathrm{cqve}}}_{\tau }(v)$			
*τ*	*v*	*h*	*n*	Bias	esd	sd	cp	Bias	esd	sd	cp
0.1	0.6	0.15	1000	0.031	0.274	0.259	0.926	0.007	0.062	0.058	0.942
			1500	0.020	0.220	0.213	0.932	0.006	0.051	0.049	0.935
		0.2	1000	0.021	0.231	0.218	0.930	0.006	0.058	0.055	0.939
			1500	0.003	0.185	0.176	0.925	0.002	0.047	0.046	0.932
		$h_{\text{opt}}$	1000	0.014	0.198	0.188	0.932	0.004	0.053	0.051	0.938
			1500	0.004	0.175	0.153	0.952	0.003	0.046	0.042	0.946
	0.8	0.15	1000	0.015	0.270	0.249	0.937	0.012	0.085	0.079	0.958
			1500	0.019	0.220	0.211	0.930	0.010	0.069	0.067	0.946
		0.2	1000	0.012	0.236	0.212	0.934	0.009	0.081	0.076	0.958
			1500	0.012	0.189	0.178	0.916	0.004	0.066	0.063	0.942
		$h_{\text{opt}}$	1000	0.016	0.212	0.195	0.922	0.006	0.077	0.073	0.952
			1500	0.018	0.175	0.165	0.926	0.005	0.065	0.058	0.954
0.3	0.6	0.15	1000	0.022	0.238	0.235	0.938	0.005	0.055	0.051	0.945
			1500	0.014	0.194	0.185	0.930	0.004	0.044	0.045	0.938
		0.2	1000	0.012	0.200	0.198	0.935	0.004	0.051	0.048	0.952
			1500	0.014	0.165	0.154	0.941	0.004	0.042	0.041	0.951
		$h_{\text{opt}}$	1000	0.007	0.171	0.167	0.942	0.003	0.047	0.043	0.962
			1500	0.001	0.149	0.140	0.946	0.001	0.039	0.037	0.942
	0.8	0.15	1000	0.005	0.238	0.240	0.927	0.008	0.073	0.069	0.959
			1500	0.021	0.199	0.188	0.939	0.007	0.060	0.059	0.940
		0.2	1000	0.002	0.202	0.198	0.935	0.005	0.069	0.066	0.958
			1500	0.014	0.166	0.162	0.948	0.006	0.057	0.055	0.948
		$h_{\text{opt}}$	1000	0.002	0.177	0.176	0.924	0.003	0.066	0.063	0.950
			1500	0.008	0.154	0.146	0.956	0.002	0.055	0.051	0.962

esd, estimated standard deviation; sd, standard deviation; cp, coverage probability.

In the Supplementary Material further figures display the estimated curves of $qve_{\tau }(v)$ and $\mathrm{cqv}e_{\tau }(v)$ with the pointwise and simultaneous confidence bands under models M1–M4 for $h_{\text{opt}}$ and n=1000. It can be seen that the estimated curves are close to their true curves and that the confidence bands cover the entire true curves. The results for other settings are similar. In addition, in the Supplementary Material we also display the estimated curves of $\beta_{1\tau }^{*}(v)$ and $\beta_{2\tau }^{*}(v)$ with the pointwise confidence intervals under models M1–M4 for $h_{\text{opt}}$ and n=1000. The results suggest that the estimators ${\hat{\beta }}_{1\tau }(v)$ and ${\hat{\beta }}_{2\tau }(v)$ are nearly unbiased, and that the confidence intervals also cover the entire true curves.

To investigate the performance of the test procedures, we also provide the empirical sizes and powers of the test statistics $T_{11},\;T_{12}(\tau ),\;T_{21}$ and $T_{22}(\tau )$ at a significance level of 0.05. The results are reported in Table 2. We see that the empirical sizes of all tests are close to the nominal 5% level, and all tests have reasonable powers to detect deviations from the null hypothesis. The powers of all tests increase as the simulation model moves from M2 to M4. In addition, the tests with the optimal bandwidth achieve higher powers than those with *h* = 0.15 and 0.2. Furthermore, we observe that the estimated standard deviation of ${\hat{\mathrm{cqve}}}_{\tau }(v)$ with *h* = 0.2 is smaller than that with h=0.15, which suggests that the empirical powers of the tests increase as the bandwidth varies from 0.15 to 0.2. This is because a larger bandwidth usually leads to a smaller esd of ${\hat{\mathrm{cqve}}}_{\tau }(v),$ but the biases remain approximately the same, resulting in increased power for the larger bandwidth. Such a phenomenon was also observed by Sun et al. (2009) under the mark-specific proportional hazard model, which may be associated with the convergence rate of the normalized ${\hat{\mathrm{cqve}}}_{\tau }(v)$ to a Gaussian process.

**Table 2:** Empirical sizes and powers of tests $T_{11},\;T_{12}(\tau ),\;T_{21}$ and $T_{22}(\tau )$ at the nominal level 0.05. The reported values are in percentages

Model	*h*	*n*	$T_{11}$	$T_{12}(0.1)$	$T_{12}(0.2)$	$T_{12}(0.3)$	$T_{21}$	$T_{22}(0.1)$	$T_{22}(0.2)$	$T_{22}(0.3)$
M1	0.15	1000	8.0	2.7	2.7	3.4	3.9	4.3	3.4	3.2
		1500	7.6	4.0	3.2	3.6	4.2	3.7	3.5	3.5
	0.2	1000	8.3	3.0	2.7	3.3	4.4	4.1	4.0	3.3
		1500	7.4	3.5	4.2	4.2	5.1	5.2	3.7	3.6
	$h_{\text{opt}}$	1000	7.0	3.8	2.6	3.4	5.8	3.6	4.0	3.6
		1500	7.2	2.2	3.4	2.2	3.8	4.2	4.6	5.0
M2	0.15	1000	53.2	73.1	82.0	82.2	3.1	5.6	4.4	4.1
		1500	76.1	89.4	94.5	95.1	2.9	4.4	5.5	5.4
	0.2	1000	61.7	74.0	84.8	85.9	2.9	4.4	4.2	4.9
		1500	86.1	90.9	96.6	96.9	4.7	6.0	5.7	5.1
	$h_{\text{opt}}$	1000	73.6	76.4	85.8	90.0	3.0	5.2	4.4	5.0
		1500	88.4	92.4	97.6	97.0	3.6	5.4	6.4	6.0
M3	0.15	1000	75.1	74.3	82.4	82.1	13.4	26.9	41.2	47.8
		1500	95.1	90.6	95.4	96.5	31.5	39.2	57.1	68.0
	0.2	1000	89.0	77.6	89.9	91.5	31.9	32.6	55.0	65.4
		1500	99.1	92.2	97.9	98.4	57.1	46.6	72.5	82.9
	$h_{\text{opt}}$	1000	96.2	82.6	91.4	94.0	53.0	42.2	67.2	77.8
		1500	99.4	95.0	99.2	98.5	69.2	56.8	80.2	88.6
M4	0.15	1000	98.1	94.0	98.7	97.1	31.5	49.8	69.0	72.6
		1500	100	98.5	100	99.9	61.7	68.3	85.6	91.0
	0.2	1000	99.6	95.6	99.1	98.7	57.1	58.6	78.8	84.9
		1500	100	98.7	99.9	99.9	82.8	79.7	93.1	97.1
	$h_{\text{opt}}$	1000	99.8	96.8	99.3	99.8	69.2	73.5	90.3	94.7
		1500	100	99.2	100	100	91.0	85.4	96.2	98.6

## 5 Real data analysis

For illustration purposes, we applied the proposed method to a dataset from the first HIV vaccine efficacy trial. The trial was carried out in North America and The Netherlands, and 5403 HIV-negative volunteers at risk were enrolled for acquiring HIV infection (Flynn et al., 2005). Volunteers were randomly assigned to receive either a recombinant glycoprotein 120 vaccine, aidsvax, or a placebo in a 2:1 ratio, and were monitored for HIV infection at semi-annual HIV testing visits for 36 months. The vaccine may only provide protection for HIV strains genetically similar to the HIV virus or viruses represented in the vaccine. The similarity between the infecting virus and the virus contained in the vaccine construct was measured by the genetic distance, which was defined as the percent mismatch of amino acids between two aligned HIV sequences. Since the HIV-gp120 region contained neutralizing epitopes that potentially induced anti-HIV antibody responses that prevented HIV infection (Wyatt et al., 1998), we defined mark *V* as the percent mismatch of amino acids in the whole gp120 region (581 amino acids long), where all possible mismatches of particular pairs of amino acids, e.g., A versus C, were weighted by the estimated probability of interchange; see the 2005 technical report from the University of Washington by D. C. Nickle *et al.* During the trial, 368 individuals were infected with HIV, but 32 individuals had missing marks. Since our proposed method cannot be directly applied to handle missing data, we removed the 32 individuals with a missing mark, and focused on the analysis of the remaining 336 samples, as in Sun et al. (2009) and Han et al. (2017). Each of the remaining 336 samples, 217 vaccine and 119 placebo, had a unique mark, and mark *V* ranged from 0.059 to 0.261.

Following Sun et al. (2009) and Han et al. (2017), we considered three covariates: treatment indictor, $Z_{1},$ taking value 1 if the volunteer was in the vaccine group and 0 otherwise; age at enrolment, $Z_{2},$ ranging from 18–62 years with a median of 36; and behavioural risk score, $Z_{3},$ taking values 0–7, as defined in Flynn et al. (2005). According to the preliminary analysis as in Fig. 1, we assumed that the data can be described by model (1) on $\left[\tau_{0},\tau_{U}]=[0.05,0.15\right]$ and [a,b]=[0.1,0.2]. We used the Epanechnikov kernel, and the grid points were kept the same as those in the simulation studies. We used the five-fold cross-validation method to select the optimal bandwidth, and the optimal bandwidth $h_{\text{opt}}=0.04$, as shown in Fig. S10 of the Supplementary Material.

The estimated curves for $\beta_{1\tau }^{*}(v),\beta_{2\tau }^{*}(v)$ and $\beta_{3\tau }^{*}(v)$ with τ=0.05,0.1 and 0.15 and their pointwise confidence bands are given in the Supplementary Material. We found that age and behaviour risk score had significant effects on the risk of infection, but the vaccine did not seem to be significantly related to the risk of HIV infection. For example, age had a significant negative effect for the mark larger than 0.14 at τ=0.05, but was nonsignificant over the whole mark interval for τ=0.1 and 0.15. The behaviour risk score had a negative decreasing effect over the whole mark interval at τ=0.05, while it showed a negative inverted U-shaped pattern for τ=0.1 and 0.15. For comparison, we also analysed the data with the method of Peng & Huang (2008) that does not consider the mark, and the comparison results are given in the Supplementary Material. It can be seen that the curves estimated by Peng and Huang’s method were very different from ours. In particular, Peng and Huang’s method showed that the covariate effects did not change with the quantiles, for example, the estimated values for $\beta_{1\tau }^{*}(v)$ were between 0.019 914 62 and 0.019 914 94, while our method indicated that the covariate effects varied with the quantiles.

**Fig. 2:**
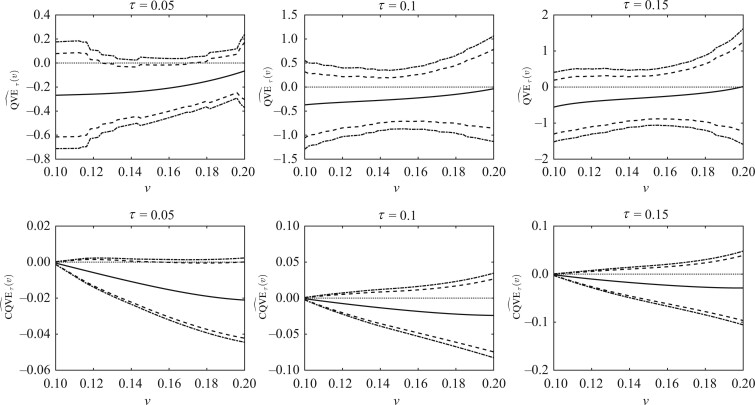
Vaccine trial data analysis: The estimated curves of $qve_{\tau }(v)$ and $\mathrm{cqv}e_{\tau }(v)$ with τ=0.05,0.1 and 0.15. The solid lines are the estimated functions, the dashed lines are the pointwise 95% confidence intervals, and the dash-dotted lines are the simultaneous 95% confidence bands.

The test statistic $T_{11}$ for *H*_10_ versus *H*_11_ yielded a *p*-value of 0.193. The *p*-value of $T_{12}(\tau )$ was larger than 0.798 for testing *H*_10_ against $H_{12}.$ These results suggested that the vaccine had no significant efficacy against HIV; see Fig. 2 for the plots of ${\hat{\mathrm{qve}}}_{\tau }(v)$ and ${\hat{\mathrm{cqve}}}_{\tau }(v).$ In addition, we conducted the tests to evaluate whether the vaccine efficacy varied with the mark. The *p*-value for testing *H*_20_ versus *H*_21_ was 0.728 for $T_{21},$ while the *p*-value for testing against *H*_21_ was larger than 0.793 for $T_{22}(\tau ).$ This indicated that the vaccine efficacy had no varying tendency on the considered mark interval. These results were consistent with those obtained by Han et al. (2017).

## Supplementary Material

asad039_Supplementary_Material

## Supplementary material

The Supplementary Material includes the proofs of Theorems 1–3, confidence bands for vaccine efficacy, a discussion on multivariate continuous marks, and additional simulation and application results.
