# Supplementary material for: A mark-specific quantile regression model
Source: Biometrika. 2023 Jun 20;111(1):255–72. doi: 10.1093/biomet/asad039 (PMC11212524; doi:10.1093/biomet/asad039)
Supplement: asad039_Supplementary_Material [file asad039_supplementary_material.pdf]

Biometrika (2023), **xx**, x, *pp*. 1–8
Printed in Great Britain

Supplementary material for "Mark-specific quantile regression model"

BY LIANQIANG QU

School of Mathematics and Statistics, Central China Normal University, Wuhan, Hubei 430079, China

LIUQUAN SUN

Institute of Applied Mathematics, Academy of Mathematics and Systems Science, Chinese Academy of Sciences, Beijing, 100190, China

slq@amt.ac.cn

AND YANQING SUN

Department of Mathematics and Statistics, University of North Carolina at Charlotte, Charlotte, U.S.A.

1. PROOFS OF THEOREMS 1-3

LEMMA S1. *Assume that* $\psi_n(\xi, v, \tau)$ *are random functions and* $\psi(\xi, v, \tau)$ *is a fixed function of* $(\xi, v, \tau) \in \Theta \times \mathcal{B}, \Theta \in \mathbb{R}^{p+1}, \mathcal{B} = [a, b] \times [\tau_0, \tau_U]$. *Let* $\beta^*_\tau(v)$ *be a fixed function of* $(v, \tau) \in \mathcal{B}$ *taking values in* Θ. *Suppose that* $\sup_{(\xi,v,\tau)} \|\psi_n(\xi, v, \tau) - \psi(\xi, v, \tau)\| = o_p(1)$, *and that for every* $\epsilon > 0$, *there exists an* $\eta > 0$ *such that* $\inf_{\|\xi-\beta^*_\tau(v)\|>\epsilon} \|\psi(\xi, v, \tau)\| > \eta$, *and* $\psi(\beta^*_\tau(v), v, \tau) = 0$ *for* $(v, \tau) \in \mathcal{B}$. *Then for any sequence of estimators* $\hat\beta_\tau(v)$ *with* $\psi_n(\hat\beta_\tau(v), v, \tau) = o_p(1)$ *uniformly in* $(v, \tau) \in \mathcal{B}$, *we have* $\|\hat\beta_\tau(v) - \beta^*_\tau(v)\| = o_p(1)$ *uniformly in* $(v, \tau) \in \mathcal{B}$.

Proof. It follows the proof of Lemma 1 in Sun et al. (2009) with the functions $Q_n(\xi, v, \tau) = -\|\psi_n(\xi, v, \tau)\|^2$ and $Q(\xi, v, \tau) = -\|\psi(\xi, v, \tau)\|^2$. □

Proof of Theorem 1. We begin with the following decomposition

$$\|\hat S_n(\xi, \tau, v) - U_n(\xi, \tau, v)\| \le \|\hat S_n(\xi, \tau, v) - \hat U_n(\xi, \tau, v)\| + \|\hat U_n(\xi, \tau, v) - U_n(\xi, \tau, v)\|, \tag{A.1}$$

where

$$\hat S_n(\xi, \tau, v) = \frac{1}{n}\sum_{i=1}^n Z_i\left[\int_0^1\int_0^L \frac{1}{\hat G(t)}\Phi\Big\{\frac{Z_i^\top\xi - \log t}{(nh)^{-1/2}\gamma_i}\Big\}K_h(u-v)N_i(dt, du) - \tau\right],$$

$$\hat U_n(\xi, \tau, v) = \frac{1}{n}\sum_{i=1}^n Z_i\left[\int_0^1\int_0^L \frac{1}{\hat G(t)}I\{\log t \le Z_i^\top\xi\}K_h(u-v)N_i(dt, du) - \tau\right],$$

$$U_n(\xi, \tau, v) = \frac{1}{n}\sum_{i=1}^n Z_i\left[\int_0^1\int_0^L \frac{1}{G(t)}I\{\log t \le Z_i^\top\xi\}K_h(u-v)N_i(dt, du) - \tau\right].$$

© 2020 Biometrika Trust

For the first term on the right hand of (A.1), we have

$$\|\hat{S}_n(\xi,\tau,v)-\hat{U}_n(\xi,\tau,v)\| \leq \left\| \frac{1}{n}\sum_{i=1}^{n} Z_i \int_0^1\int_0^L K_h(u-v)\frac{N_i(dt,du)}{\hat{G}(t)} \right\| \times \left(\frac{1}{n}\sum_{i=1}^{n} \left| I(Z_i^\top\xi>0)-\Phi\Big\{\frac{Z_i^\top\xi-\log X_i}{(nh)^{-1/2}\gamma_i}\Big\}\right|^2\right)^{1/2}. \quad (A.2)$$

By Conditions 1-6 and the facts that $\sup_{t\in[0,L]}|\hat{G}(t)-G(t)|=o(1)$ almost surely, the first term on the right hand side of (A.2) converges uniformly in v to its expectation almost surely, which is finite. For any $\vartheta>0$, $n^{-1}\sum_{i=1}^n I(|Z_i^\top\xi|<\vartheta)$ converges almost surely to $P(|Z^\top\xi|<\vartheta)$ uniformly in $\|\xi\|\leq M$, due to the fact that $\{I(|z^\top\xi|<\vartheta):\|\xi\|\leq M\}$ is a VC-class with finite VC-index (Kosorok, 2008), where M is a positive constant specified later. The second term on the right hand side of (A.2) converges almost surely to zero uniformly in (v,τ,ξ) by Lemma 4 of Horowitz (1992). Thus, $\sup_{(v,\tau,\xi)}\|\hat{S}_n(\xi,\tau,v)-\hat{U}_n(\xi,\tau,v)\|=o(1)$ almost surely. Similarly, we can show that the second term on the right hand side of (A.1) also converges to zero almost surely uniformly in (v,τ,ξ). Therefore, by (A.1), we have that almost surely uniformly in (v,τ,ξ),

$$\|\hat{S}_n(\xi,\tau,v)-U_n(\xi,\tau,v)\|=o(1). \quad (A.3)$$

Define

$$\theta_n(\xi,v)=\frac{1}{n}\sum_{i=1}^n Z_i\int_0^v\int_0^L \frac{1}{G(t)}I\{\log t\leq Z_i^\top\xi\}N_i(dt,du).$$

Let $\mathcal{F}_1=\{Z_i\int_0^v\int_0^L I(\log t\leq Z_i^\top\xi)/G(t)N_i(dt,du):\|\xi\|\leq M, v\in[a,b]\}$ and $\mathcal{F}_2=\{Z_i\tau:\tau\in[\tau_0,\tau_U]\}$. It can be shown that the classes $\mathcal{F}_1$ and $\mathcal{F}_2$ are P-Donsker using the Donsker preservation properties (Corollary 9.32, Kosorok (2008)). Thus, $\theta_n(\xi,v)=\theta(\xi,v)+O_p(n^{-1/2})$ uniformly in (v,ξ), and $n^{-1}\sum_{i=1}^n Z_i\tau=\tau E(Z)+O_p(n^{-1/2})$ uniformly in τ, where

$$\theta(\xi,v)=E\Big[Z\int_0^v\int_0^L\frac{1}{G(t)}I\{\log t\leq Z^\top\xi\}N(dt,du)\Big].$$

Note that $U_n(\xi,\tau,v)=\int_0^1 K_h(u-v)\theta_n(\xi,du)-n^{-1}\sum_{i=1}^n Z_i\tau$. It then follows that uniformly in (v,τ,ξ),

$$U_n(\xi,\tau,v)=U(\xi,\tau,v)+O_p\big((nh^2)^{-1/2}\big), \quad (A.4)$$

where $U(\xi,\tau,v)=E[Z(F_v\{\exp(Z^\top\xi)|Z\}-\tau)]$ is a nonrandom function. By (A.3) and (A.4), we have that uniformly in (v,τ,ξ),

$$\begin{aligned}&\|\hat{S}_n(\xi,\tau,v)-U(\xi,\tau,v)\|\\ \leq&\|\hat{S}_n(\xi,\tau,v)-U_n(\xi,\tau,v)\|+\|U_n(\xi,\tau,v)-U(\xi,\tau,v)\|=o_p(1). \quad (A.5)\end{aligned}$$

Let $\epsilon>0$ be any constant such that $\sup_{(v,\tau)\in\mathcal{B}}\|\beta_\tau(v)-\beta_\tau^*(v)\|<\epsilon$. For any $w\in\mathbb{R}^{p+1}$ satisfying $\|w\|=1$, $w^\top U(\beta_\tau^*(v)+w\epsilon_0,\tau,v)$ is increasing in ϵ_0, which implies that for any

$\epsilon_0 > \epsilon > 0$, $w^\top[U(\beta_\tau^*(v) + w\epsilon_0, \tau, v) - U(\beta_\tau^*(v), \tau, v)] \geq 0$. Then, we have

$$\begin{aligned}\|w\|\|U(\beta_\tau^*(v) + w\epsilon_0, \tau, v) - U(\beta_\tau^*(v), \tau, v)\| \geq& |w^\top[U(\beta_\tau^*(v) + w\epsilon_0, \tau, v) - U(\beta_\tau^*(v), \tau, v)]| \\ \geq& |w^\top[U(\beta_\tau^*(v) + w\epsilon, \tau, v) - U(\beta_\tau^*(v), \tau, v)]| \\ \geq& w^\top A(\beta_\tau^*(v) + w\bar{\epsilon}, v)w\epsilon > \bar{\kappa}\epsilon,\end{aligned}$$

where $A(\xi, v) = E\{ZZ^\top \exp(Z^\top\xi)f(\exp(Z^\top\xi), v|Z)\}$, $\bar{\epsilon} \in [0, \epsilon]$ and $\bar{\kappa} > 0$ is some constant. Here the first inequality holds due to the Cauchy-Schwarz inequality, and the last one follows from Condition 4 and the continuity of $A(\xi, v)$. Therefore,

$$\inf_{\|\beta_\tau(v) - \beta_\tau^*(v)\| > \epsilon} \|U(\beta_\tau(v), \tau, v) - U(\beta_\tau^*(v), \tau, v)\| > \bar{\kappa}\epsilon. \qquad \text{(A.6)}$$

By the definitions of $\hat{\beta}_\tau(v)$ and $\beta_\tau^*(v)$, we have $\hat{S}_n(\hat{\beta}_\tau(v), \tau, v) = 0$ almost surely and $U(\beta_\tau^*(v), \tau, v) = 0$. It then follows from (A.5) that with probability tending to one uniformly in (v, τ),

$$\begin{aligned}&\|U(\hat{\beta}_\tau(v), \tau, v) - U(\beta_\tau^*(v), \tau, v)\| \\ =&\|\hat{S}_n(\hat{\beta}_\tau(v), \tau, v) - \{\hat{S}_n(\hat{\beta}_\tau(v), \tau, v) - U(\hat{\beta}_\tau(v), \tau, v)\}\| < \bar{\kappa}\epsilon/2.\end{aligned}$$

This, together with (A.6), implies that $\|\hat{\beta}_\tau(v) - \beta_\tau^*(v)\| \leq \epsilon$ with probability tending to one. By Condition 2, there exists some constant M_0 such that $\|\beta_\tau^*(v)\| \leq M_0$. These facts imply that there exists an $M > M_0$ such that $\|\hat{\beta}_\tau(v)\| \leq \|\beta_\tau^*(v)\| + \|\hat{\beta}_\tau(v) - \beta_\tau^*(v)\| \leq M$ with probability tending to one, that is, $P(\|\hat{\beta}_\tau(v)\| \leq M, (v, \tau) \in \mathcal{B}) > 1 - \alpha$ for every $\alpha > 0$ and all large n. Then, by Lemma 1 and taking $\Theta = \{\xi \in \mathbb{R}^{p+1} : \|\xi\| \leq M\}$, we have that for every $\epsilon > 0$,

$$\begin{aligned}&P\Big(\sup_{(v,\tau)\in\mathcal{B}} \|\hat{\beta}_\tau(v) - \beta_\tau^*(v)\| > \epsilon\Big) \\ \leq&\alpha + P\Big(\sup_{(v,\tau)\in\mathcal{B}} \|\hat{\beta}_\tau(v) - \beta_\tau^*(v)\| > \epsilon, \|\hat{\beta}_\tau(v)\| \leq M, (v, \tau) \in \mathcal{B}\Big) \to \alpha,\end{aligned}$$

as $n \to \infty$. Since α can be arbitrarily small, we have

$$\sup_{(v,\tau)\in\mathcal{B}} \|\hat{\beta}_\tau(v) - \beta_\tau^*(v)\| = o_p(1).$$

This completes the proof.

Proof of Theorem 2. We begin with the following decomposition

$$\sqrt{nh}\hat{S}_n\{\beta_\tau^*(v)\} = \sqrt{nh}S_n\{\beta_\tau^*(v)\} + \sqrt{nh}\big[\hat{S}_n\{\beta_\tau^*(v)\} - S_n\{\beta_\tau^*(v)\}\big], \qquad \text{(A.7)}$$

where

$$S_n(\xi, \tau, v) = \frac{1}{n}\sum_{i=1}^n Z_i\left[\int_0^1\int_0^L \frac{1}{G(t)}\Phi\Big\{\frac{Z_i^\top\xi - \log t}{(nh)^{-1/2}\gamma_i}\Big\}K_h(u - v)N_i(dt, du) - \tau\right].$$

By Conditions 1-3 and 5, we have

$$\begin{aligned}
&E\big[S_n\{\beta_\tau^*(v)\} - U_n\{\beta_\tau^*(v)\}\big] \\
=&E\Bigg(Z\Bigg[\Phi\Big(\frac{Z^\top\beta_\tau^*(v) - \log T}{(nh)^{-1/2}\gamma}\Big) - I\big(\log T \le Z^\top\beta_\tau^*(v)\big)\Bigg]K_h(U-v)\Bigg) \\
=&\int \frac{z\gamma}{(nh)^{1/2}}\Big[\Phi\big(u\big) - I(u\ge 0)\Big]f\Big(\exp\{z^\top\beta_\tau^*(v) - u\gamma(nh)^{-1/2}\}, v|z\Big) \\
&\qquad\times f_Z(z)\exp\{z^\top\beta_\tau^*(v) - u\gamma(nh)^{-1/2}\}dudz + O(h^2),
\end{aligned} \tag{A.8}$$

where $f(t,v|z)$ is the conditional density function of (T,V) given $Z=z$, and $f_Z(z)$ is the density function of Z. Note that

$$\begin{aligned}
\int_{-\infty}^{\infty}[\Phi(u) - I(u\ge 0)]du &= \int_0^\infty(\Phi(u)-1)du + \int_{-\infty}^0\Phi(u)du \\
&= \int_0^\infty(\Phi(u)-1)du + \int_0^\infty\Phi(-u)du \\
&= \int_0^\infty(\Phi(u)-1)du + \int_0^\infty(1-\Phi(u))du = 0.
\end{aligned}$$

By the Taylor expansion and Conditions 1-3 and 5, we have that the first term on the right hand side of (A.8) is

$$\begin{aligned}
&\int \frac{z\gamma}{(nh)^{1/2}}f(\exp\{z^\top\beta_\tau^*(v)\}, v|z)f_Z(z)\exp\{z^\top\beta_\tau^*(v)\}dz \\
&\qquad\times\int\big[\Phi\big(u\big) - I(u\ge 0)\big]du + O\Big(\frac{1}{nh}\Big) = O\Big(\frac{1}{nh}\Big).
\end{aligned}$$

Thus,

$$E\big[S_n\{\beta_\tau^*(v)\} - U_n\{\beta_\tau^*(v)\}\big] = O((nh)^{-1} + h^2).$$

Moreover, we can show

$$\mathrm{Var}\big[S_n\{\beta_\tau^*(v)\} - U_n\{\beta_\tau^*(v)\}\big] = O\big((nh)^{-3/2}\big).$$

Then by Condition 6, the first term on the right hand side of (A.7) is asymptotic equivalent to

$$\sqrt{\frac{h}{n}}\sum_{i=1}^n Z_i\Bigg[\int_0^1\int_0^L\frac{1}{G(t)}I\big\{\log t\le Z_i^\top\beta_\tau^*(v)\big\}K_h(u-v)N_i(dt,du) - \tau\Bigg] + o_p(1).$$

Therefore, $\sqrt{nh}S_n\{\beta_\tau^*(v)\}$ converges in distribution to a zero-mean normal random vector with covariance matrix $D(v,\tau)$, where

$$D(v,\tau) = \nu_0 E[ZZ^\top I(\log T\le Z^\top\beta_\tau^*(v))G(T)^{-1}g(v|Z,T)].$$

By Theorem 2.1 of Pepe (2003), we have that uniformly in $t\in[0,L]$,

$$\sqrt{n}\{\hat{G}_n(t) - G(t)\} = -\frac{1}{\sqrt{n}}\sum_{i=1}^n G(t)\int_0^t\frac{dM_i^C(s)}{\bar{y}(s)} + o_p(1).$$

For the second term on the right hand side of (A.7), we get

$$
\begin{aligned}
&\sqrt{nh}\big[\hat{S}_n\{\beta^*_\tau(v)\} - S_n\{\beta^*_\tau(v)\}\big]\\
=&\sqrt{\frac{h}{n}}\sum_{j=1}^n \int_0^1\int_0^L\int_0^t \frac{dM_j^C(s)}{\bar{y}(s)G(t)}\frac{1}{n}\sum_{i=1}^n Z_i\Phi\Big\{\frac{Z_i^\top\beta^*_\tau(v)-\log t}{(nh)^{-1/2}\gamma_i}\Big\}K_h(u-v)N_i(dt,du)+o_p(1)\\
=&\sqrt{\frac{h}{n}}\sum_{i=1}^n \int_0^L E\Big[ZI\Big(s\le T\le \exp\{Z^\top\beta^*_\tau(v)\}\Big)g(v|Z,T)\Big]\frac{dM_i^C(s)}{\bar{y}(s)}+o_p(1).
\end{aligned}
$$

It is of order $O_p(\sqrt{h})$ uniformly in (v,τ). Next, by the Taylor expansion of $\hat{S}_n\{\beta_\tau(v)\}$ at $\beta^*_\tau(v)$, we obtain

$$
\sqrt{nh}[\hat{S}_n\{\hat{\beta}_\tau(v)\} - \hat{S}_n\{\beta^*_\tau(v)\}] = \hat{A}\{\breve{\beta}_\tau(v)\}\sqrt{nh}\{\hat{\beta}_\tau(v)-\beta^*_\tau(v)\}+o_p(1),
$$

where $\breve{\beta}_\tau(v)$ is on the line segment between $\hat{\beta}_\tau(v)$ and $\beta^*_\tau(v)$, and

$$
\hat{A}(\xi) = \frac{1}{n}\sum_{i=1}^n \frac{Z_iZ_i^\top}{(nh)^{-1/2}\gamma_i}\int_0^1\int_0^L \frac{1}{G(t)}\phi\Big\{\frac{Z_i^\top\xi-\log t}{(nh)^{-1/2}\gamma_i}\Big\}K_h(u-v)N_i(dt,du).
$$

Note that $\hat{A}\{\beta^*_\tau(v)\}$ converges to $E[ZZ^\top\exp\{Z^\top\beta^*_\tau(v)\}f(\exp\{Z^\top\beta^*_\tau(v)\},v|Z)]$ with probability tending to 1 uniformly in (v,τ). This, together with the fact that $\hat{\beta}_\tau(v)$ uniformly converges in probability to $\beta^*_\tau(v)$, implies that $\hat{A}\{\breve{\beta}_\tau(v)\}$ converges to $A_\tau(v)$ with probability tending to 1 uniformly in (v,τ) and hence is nonsingular by Condition 4. Thus, $\sqrt{nh}\{\hat{\beta}_\tau(v)-\beta^*_\tau(v)\}$ converges weakly to a zero-mean normal random vector with covariance matrix $A_\tau(v)^{-1}D(v,\tau)A_\tau(v)^{-1}$. This completes the proof.

Proof of Theorem 3. By the proof of Theorem 2, we have that for any $v\in[a,b]$,

$$
\begin{aligned}
\sqrt{n}\int_a^v\{\hat{\beta}_\tau(u)-\beta^*_\tau(u)\}du =& -\frac{1}{\sqrt{n}}\sum_{i=1}^n\int_a^v A_\tau(u)^{-1}\tilde{\varphi}_{1i}(u,\tau)du\\
&-\frac{1}{\sqrt{n}}\sum_{i=1}^n\int_a^v A_\tau(u)^{-1}\tilde{\varphi}_{2i}(u,\tau)du+o_p(1),
\end{aligned}
\tag{A.9}
$$

where

$$
\begin{aligned}
\tilde{\varphi}_{1i}(v,\tau) &= Z_i\Big[\int_0^1\int_0^L I\big\{\log t\le Z_i^\top\beta^*_\tau(v)\big\}K_h(u-v)\frac{N_i(dt,du)}{G(t)}-\tau\Big],\\
\tilde{\varphi}_{2i}(v,\tau) &= \int_0^L E\Big[ZI\Big(s\le T\le\exp\{Z^\top\beta^*_\tau(v)\}\Big)g(v|Z,T)\Big]\bar{y}(s)^{-1}dM_i^C(s).
\end{aligned}
$$

By Conditions 5 and 6, we can show

$$
\begin{aligned}
&\frac{1}{\sqrt{n}}\sum_{i=1}^n\int_a^v A_\tau(u)^{-1}\tilde{\varphi}_{1i}(u,\tau)du\\
=&\frac{1}{\sqrt{n}}\sum_{i=1}^n Z_i\Big[\int_a^v\int_0^L A_\tau(u)^{-1}I\big\{\log t\le Z_i^\top\beta^*_\tau(u)\big\}\frac{N_i(dt,du)}{G(t)}-\tau\int_a^v A_\tau(u)^{-1}du\Big]\\
&+o_p(1).
\end{aligned}
$$

This, together with (A.9), implies that

$$\sqrt{n}\int_a^v \{\hat\beta_\tau(u)-\beta^*_\tau(u)\}du = -\frac{1}{\sqrt{n}}\sum_{i=1}^n \Big[\varphi_{1i}(u,\tau)+\varphi_{2i}(u,\tau)\Big] + o_p(1),$$

where

$$\varphi_{1i}(v,\tau) = \Big[\int_a^v\int_0^L A_\tau(u)^{-1} I\big\{\log t \le Z_i^\top \beta^*_\tau(u)\big\}\frac{N_i(dt,du)}{G(t)} - \tau\int_a^v A_\tau(u)^{-1}du\Big] Z_i,$$

$$\varphi_{2i}(v,\tau) = \int_a^v\int_0^L A_\tau(u)^{-1}E\Big[ZI\Big(s\le T\le \exp\{Z^\top\beta^*_\tau(u)\}\Big)g(u|Z,T)\Big]\bar y(s)^{-1}dM_i^C(s)du.$$

Define $\mathcal{F}_3=\{\varphi_{1i}(v,\tau): v\in[a,b],\tau\in[\tau_0,\tau_U]\}$ and $\mathcal{F}_4=\{\varphi_{2i}(v,\tau): v\in[a,b],\tau\in[\tau_0,\tau_U]\}$. By Condition 2, we can show that the classes $\mathcal{F}_3$ and $\mathcal{F}_4$ are P-Donsker. Furthermore, $\varphi_{1i}(v,\tau)$ and $\varphi_{2i}(v,\tau)$ are independent to each other, due to the fact that the censoring time C is independent of (T,V) given Z. Thus, $\sqrt{n}\{\hat B_\tau(u)-B^*_\tau(u)\}$ converges weakly to a mean zero Gaussian process with covariance matrix $E\{\varphi_1(v_1,\tau_1)\varphi_1(v_2,\tau_2)^\top+\varphi_2(v_1,\tau_1)\varphi_2(v_2,\tau_2)^\top\}$. This completes the proof.

2. CONFIDENCE BANDS FOR VACCINE EFFICACY

We only focus on the construction of confidence bands for the mark-specific quantile-type vaccine efficacy. The confidence bands for the regression coefficients can be obtained similarly. Under model (1), the mark-specific quantile-type vaccine efficacy can be expressed as $\text{QVE}_\tau(v)=\exp\{\beta^*_{1\tau}(v)\}-1$, which can be consistently estimated by $\widehat{\text{QVE}}_\tau(v)=\exp\{\hat\beta_{1\tau}(v)\}-1$. It follows from Theorem 2 that $(nh)^{1/2}\{\widehat{\text{QVE}}_\tau(v)-\text{QVE}_\tau(v)\}$ converges in distribution to $N(0,\sigma^2_{1\tau}(v)\exp\{2\beta^*_{1\tau}(v)\})$ for each given v and τ, where $\sigma^2_{1\tau}(v)$ denotes the second diagonal element of $\Omega_\tau(v)$. Let $\hat\sigma^2_{1\tau}(v)$ be the second diagonal element of $\hat\Omega_\tau(v)$. A pointwise $100(1-\alpha)\%$ confidence interval for $\text{QVE}_\tau(v)$ is given by

$$\widehat{\text{QVE}}_\tau(v)\pm(nh)^{-1/2}z_{\alpha/2}\hat\sigma_{1\tau}(v)\exp\{\hat\beta_{1\tau}(v)\},\quad (v,\tau)\in\mathcal{B},$$

where $z_{\alpha/2}$ is the upper $\alpha/2$-quantile of the standard normal distribution.

By Theorem 1, the cumulative mark-specific quantile-type vaccine efficacy $\text{CQVE}_\tau(v)$ can be consistently estimated by $\widehat{\text{CQVE}}_\tau(v)=\int_a^v\widehat{\text{QVE}}_\tau(u)du$. Note that

$$n^{1/2}\{\widehat{\text{CQVE}}_\tau(v)-\text{CQVE}_\tau(v)\}=n^{1/2}\int_a^v\Big[\exp\{\hat\beta_{1\tau}(u)\}-\exp\{\beta^*_{1\tau}(u)\}\Big]du.$$

Using some arguments similar to the proof of Theorem 3, we have that the distribution of $n^{1/2}\{\widehat{\text{CQVE}}_\tau(v)-\text{CQVE}_\tau(v)\}$ is asymptotically equivalent to that of $n^{-1/2}\sum_{i=1}^n\hat\vartheta_{0i}(\tau,v)$, where

$$\hat\vartheta_{0i}(\tau,v)=-\int_a^v\exp\{\hat\beta_{1\tau}(u)\}e_1^\top\hat A_\tau(u)^{-1}\big[\hat\varphi_{1i}(v,\tau)+\hat\varphi_{2i}(v,\tau)\big]du,$$

and $e_1=(0,1,0,\ldots,0)^\top\in\mathbb{R}^{p+1}$. Thus, a pointwise $100(1-\alpha)\%$ confidence interval for $\text{CQVE}_\tau(v)$ is given by

$$\widehat{\text{CQVE}}_\tau(v)\pm n^{-1/2}z_{\alpha/2}\hat\zeta^{1/2}_{0\tau}(v),\quad (v,\tau)\in\mathcal{B},$$

where $\hat\zeta_{0\tau}(v)=n^{-1}\sum_{i=1}^n\hat\vartheta^2_{0i}(\tau,v)$.

To construct simultaneous confidence bands for $\text{CQVE}_\tau(v)$ over $\mathcal{B}$, define

$$\mathcal{Q} = \sup_{(v,\tau)\in\mathcal{B}} |n^{1/2}\{\widehat{\text{CQVE}}_\tau(v) - \text{CQVE}_\tau(v)\}/\hat{\zeta}_{0\tau}^{1/2}(v)|.$$

Here, we propose to use the following resampling approach to obtain the percentiles of $\mathcal{Q}$ (Lin et al., 1993). Specifically, let

$$\mathcal{S}_n\{\hat{\beta}_\tau(v)\} = -n^{-1/2}\sum_{i=1}^{n} W_i \int_a^v \exp\{\hat{\beta}_{1\tau}(u)\}e_1^\top \hat{A}_\tau(u)^{-1}\big[\hat{\varphi}_{1i}(v,\tau) + \hat{\varphi}_{2i}(v,\tau)\big]du,$$

where W_i $(i = 1,\ldots,n)$ are independent standard normal variables and are independent of the observed data. According to the arguments of Lin et al. (1993), the distribution $n^{1/2}\{\widehat{\text{CQVE}}_\tau(v) - \text{CQVE}_\tau(v)\}$ can be approximated by the conditional distribution of $\mathcal{S}_n\{\hat{\beta}_\tau(v)\}$ given the observed data. Thus, the distribution of $\mathcal{Q}$ can be approximated by the conditional distribution of $\mathcal{S}^*$ given the observed data, where

$$\mathcal{S}^* = \sup_{(v,\tau)\in\mathcal{B}} \big|\mathcal{S}_n\{\hat{\beta}_\tau(v)\}/\hat{\zeta}_{0\tau}^{1/2}(v)\big|.$$

By repeatedly generating the normal random sample W_i $(i = 1,\ldots,n)$, we can obtain the $(1-\alpha)$th percentile $z^*(\alpha)$ of the conditional distribution of $\mathcal{S}^*$. Then, a simultaneous $100(1-\alpha)\%$ confidence band for $\text{CQVE}_\tau(v)$ over $\mathcal{B}$ is given by

$$\widehat{\text{CQVE}}_\tau(v) \pm n^{-1/2}z^*(\alpha)\hat{\zeta}_{0\tau}^{1/2}(v).$$

3. MULTIVARIATE CONTINUOUS MARKS

The proposed method can be extended to the case of multivariate continuous marks. Specifically, assume that there are m continuous mark variables $(V_1,\ldots,V_m)$ taking value in the interval $[0,1]^m$, rescaled if necessary (Sun et al., 2013). Let $\tilde{v} = (v_1,\ldots,v_m)$. The conditional multivariate mark-specific cumulative incidence function can be defined as

$$F_{\tilde{v}}(t|Z) = \lim_{h_1,\ldots,h_m\to 0} P\big(T \le t,\ v_l \le V_l < v_l + h_l,\ l = 1,\ldots,m|Z\big)/(h_1\ldots h_m).$$

For $\tilde{v} \in [0,1]^m$ and $\tau \in (0,\tilde{\tau})$, the τth conditional multivariate mark-specific quantile is defined as

$$Q_{\tilde{v}}(\tau|Z) = \inf\{t : F_{\tilde{v}}(t|Z) \ge \tau\},$$

and the τth multivariate mark-specific quantile regression model speculates that

$$Q_{\tilde{v}}(\tau|Z) = \exp\{Z^\top\beta_\tau^*(\tilde{v})\},$$

where $\beta_\tau^*(\tilde{v})$ is a $(p+1)$-dimensional vector of unknown continuous functions of $\tilde{v}$ and τ. Define $N_i(t,\tilde{v}) = I(X_i \le t, \Delta_i = 1, V_{li} \le v_l,\ l = 1,\ldots,m)$, where $(V_{1i},\ldots,V_{mi})$ $(i = 1,\ldots,n)$ are independent and identically distributed copies of $(V_1,\ldots,V_m)$. Following similar arguments as in Section 2.1, we can estimate $\beta_\tau^*(\tilde{v})$ using the solution to $\hat{S}_n\{\beta_\tau(\tilde{v})\} = 0$, where

$$\begin{aligned}&\hat{S}_n\{\beta_\tau(\tilde{v})\}\\ =&\frac{1}{n}\sum_{i=1}^{n} Z_i\left[\int_0^1\cdots\int_0^1\int_0^L \frac{1}{\hat{G}(t)}\Phi\left\{\frac{Z_i^\top\beta_\tau(\tilde{v}) - \log t}{(nh)^{-1/2}\gamma_i}\right\}\mathcal{K}_{h_1,\ldots,h_m}(\tilde{u}-\tilde{v})N_i(dt,d\tilde{u}) - \tau\right],\end{aligned}$$

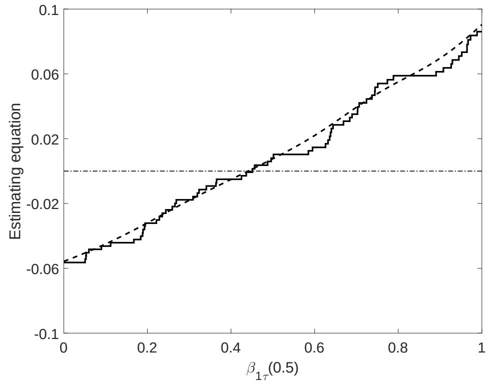

Fig. S1. Plots for the estimating equation $U_n\{\beta_\tau(v)\}$ (solid line) and its smoothed version $\hat{S}_n\{\beta_\tau(v)\}$ (dashed line).

$\mathcal{K}_{h_1,\ldots,h_m}(\tilde{u}-\tilde{v}) = \prod_{i=1}^m K_{h_l}(u_l - v_l)$, and h_l are bandwidths $(l = 1, \ldots, m)$. The asymptotic properties of the resulting estimators can be obtained in a similar manner, and will be more technically involved. Note that this approach would require large sample sizes due to the curse of dimensionality. To handle this problem, as discussed in Sun et al. (2013), we can consider a parametric model for $\beta_\tau^*(\tilde{v})$ as the first-order Taylor approximation of $\beta_\tau^*(\tilde{v})$ plus interaction terms. It would be worthwhile to further address this issue both theoretically and numerically.

REFERENCES

HOROWITZ, J. (1992). A smoothed maximum score estimator for the binary response model. *Econometrica* **63**, 505-531.

KOSOROK, M. (2008). Introduction to empirical processes and semiparametric inference. New York: Springer.

LIN, D. Y., WEI, L. J., & YING, Z. (1993). Checking the Cox model with cumulative sums of martingale-based residuals. *Biometrika* **80**, 557–572.

PEPE, M. (1991). Inference for events with dependent risks in multiple endpoint studies. *J. Amer. Statist. Assoc.* **86**, 770–778.

SUN, Y., GILBERT, P. AND MCKEAGUE, I. (2009). Proportional hazards models with continuous marks. *Ann. Statist.* **37**, 394–426.

SUN, Y., LI, M. & GILBERT, P. B. (2013). Mark-specific proportional hazards model with multivariate continuous marks and its application to HIV vaccine efficacy trials. *Biostatistics* **14**, 60–74.

4. ADDITIONAL SIMULATION AND APPLICATION RESULTS

Table S1. *Simulation results for* $\widehat{\text{QVE}}_\tau(v)$ *and* $\widehat{\text{CQVE}}_\tau(v)$ *under model M2*

				$\widehat{\text{QVE}}_\tau(v)$				$\widehat{\text{CQVE}}_\tau(v)$			
τ	v	h	n	Bias	ESD	SD	CP	Bias	ESD	SD	CP
0.1	0.6	0.15	1000	0.034	0.371	0.349	0.915	0.015	0.108	0.102	0.947
			1500	0.028	0.319	0.305	0.922	0.010	0.075	0.073	0.950
		0.2	1000	0.032	0.319	0.290	0.927	0.016	0.103	0.097	0.951
			1500	0.029	0.257	0.243	0.942	0.007	0.071	0.069	0.950
		h_{opt}	1000	0.016	0.288	0.268	0.938	0.008	0.077	0.075	0.938
			1500	0.026	0.243	0.213	0.954	0.004	0.066	0.060	0.948
	0.8	0.15	1000	0.016	0.396	0.392	0.934	0.020	0.128	0.121	0.958
			1500	0.016	0.335	0.298	0.942	0.014	0.102	0.097	0.956
		0.2	1000	0.027	0.342	0.341	0.944	0.022	0.128	0.118	0.956
			1500	0.017	0.289	0.255	0.942	0.009	0.099	0.093	0.950
		h_{opt}	1000	0.010	0.291	0.278	0.928	0.014	0.113	0.111	0.946
			1500	0.025	0.251	0.229	0.958	0.010	0.094	0.086	0.954
0.3	0.6	0.15	1000	0.021	0.308	0.295	0.930	0.010	0.085	0.078	0.953
			1500	0.007	0.288	0.276	0.938	0.008	0.068	0.063	0.970
		0.2	1000	0.038	0.261	0.251	0.940	0.025	0.078	0.075	0.942
			1500	0.005	0.247	0.231	0.950	0.007	0.064	0.058	0.966
		h_{opt}	1000	0.001	0.246	0.234	0.932	0.004	0.067	0.065	0.942
			1500	0.010	0.210	0.202	0.952	0.001	0.056	0.051	0.948
	0.8	0.15	1000	0.008	0.351	0.328	0.940	0.015	0.114	0.107	0.965
			1500	0.030	0.293	0.275	0.938	0.012	0.090	0.084	0.970
		0.2	1000	0.019	0.296	0.293	0.932	0.030	0.105	0.099	0.956
			1500	0.013	0.247	0.236	0.932	0.008	0.086	0.084	0.972
		h_{opt}	1000	-0.015	0.257	0.243	0.936	0.002	0.096	0.092	0.948
			1500	0.014	0.221	0.218	0.926	0.003	0.080	0.074	0.960

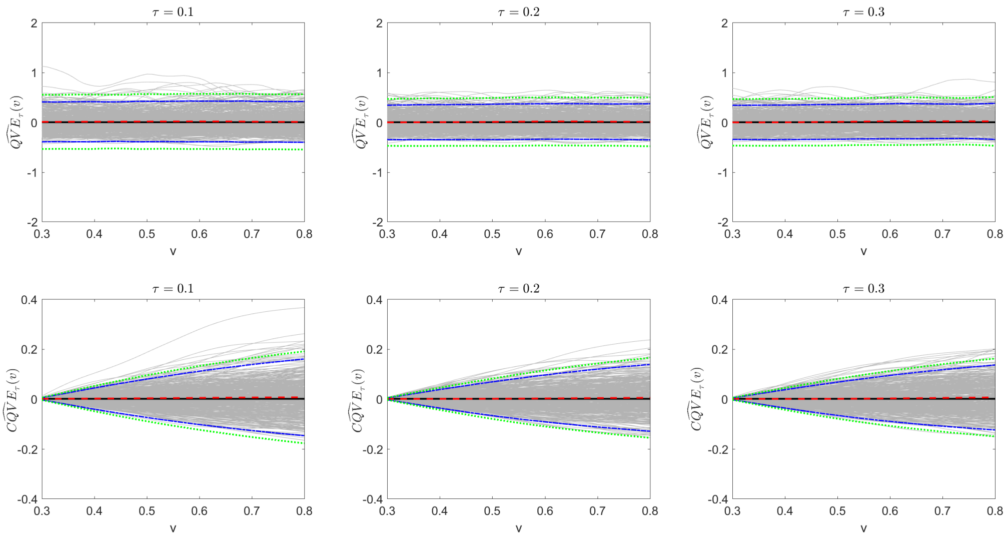

Fig. S2. Plots of the estimates for $\text{QVE}_\tau(v)$ and $\text{CQVE}_\tau(v)$ under model M1 with $n = 1000$. The bandwidth is chosen using the proposed cross-validation procedure. The sold lines are the true functions, the dashed lines are the averages of the estimates, the dash-dotted (blue) lines are the 95% pointwise confidence bands and the dotted (green) lines are the 95% simultaneous confidence bands. The gray lines are the corresponding estimates for $\text{QVE}_\tau(v)$ and $\text{CQVE}_\tau(v)$ of the 1000 random samples.

Table S2. *Simulation results for* $\widehat{\text{QVE}}_\tau(v)$ *and* $\widehat{\text{CQVE}}_\tau(v)$ *under model M3*

				$\widehat{\text{QVE}}_\tau(v)$				$\widehat{\text{CQVE}}_\tau(v)$			
τ	v	h	n	Bias	ESD	SD	CP	Bias	ESD	SD	CP
0.1	0.6	0.15	1000	0.027	0.356	0.333	0.919	0.017	0.104	0.095	0.953
			1500	0.027	0.294	0.277	0.934	0.013	0.087	0.083	0.954
		0.2	1000	0.031	0.289	0.309	0.941	0.013	0.096	0.101	0.946
			1500	0.010	0.242	0.253	0.931	0.012	0.077	0.081	0.935
		h_{opt}	1000	0.026	0.272	0.256	0.930	0.010	0.093	0.086	0.952
			1500	0.025	0.217	0.195	0.938	0.009	0.074	0.071	0.938
	0.8	0.15	1000	0.026	0.295	0.289	0.923	0.022	0.127	0.114	0.958
			1500	0.024	0.242	0.235	0.922	0.018	0.106	0.098	0.955
		0.2	1000	0.020	0.241	0.257	0.930	0.017	0.117	0.124	0.963
			1500	0.012	0.200	0.211	0.938	0.014	0.096	0.100	0.939
		h_{opt}	1000	0.022	0.234	0.217	0.948	0.015	0.116	0.111	0.948
			1500	0.027	0.192	0.177	0.944	0.014	0.095	0.090	0.954
0.3	0.6	0.15	1000	0.027	0.304	0.287	0.931	0.022	0.110	0.102	0.967
			1500	0.015	0.239	0.242	0.928	0.016	0.086	0.086	0.947
		0.2	1000	0.020	0.237	0.254	0.939	0.012	0.094	0.098	0.946
			1500	0.011	0.195	0.203	0.939	0.014	0.076	0.079	0.951
		h_{opt}	1000	0.024	0.219	0.213	0.942	0.015	0.090	0.089	0.942
			1500	0.012	0.176	0.157	0.964	0.008	0.072	0.069	0.966
	0.8	0.15	1000	0.016	0.227	0.217	0.945	0.026	0.123	0.111	0.972
			1500	0.016	0.185	0.178	0.943	0.019	0.096	0.098	0.943
		0.2	1000	0.016	0.183	0.191	0.936	0.016	0.107	0.112	0.951
			1500	0.007	0.156	0.158	0.932	0.015	0.086	0.091	0.956
		h_{opt}	1000	0.009	0.173	0.170	0.934	0.019	0.106	0.105	0.950
			1500	0.014	0.141	0.138	0.944	0.011	0.085	0.079	0.958

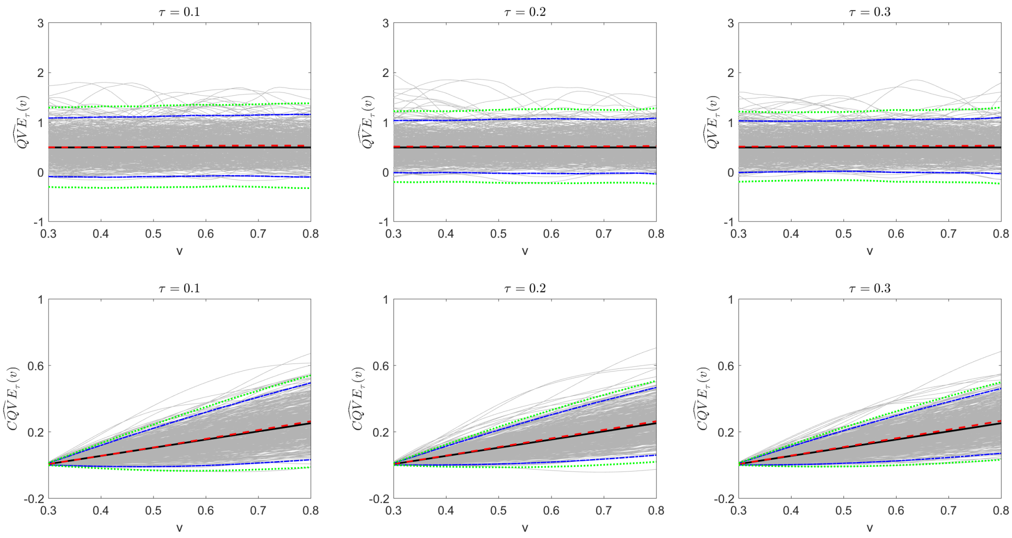

Fig. S3. Plots of the estimates for $\text{QVE}_\tau(v)$ and $\text{CQVE}_\tau(v)$ under model M2 with $n = 1000$. The bandwidth is chosen using the proposed cross-validation procedure. The sold lines are the true functions, the dashed lines are the averages of the estimates, the dash-dotted (blue) lines are the 95% pointwise confidence bands and the dotted (green) lines are the 95% simultaneous confidence bands. The gray lines are the corresponding estimates for $\text{QVE}_\tau(v)$ and $\text{CQVE}_\tau(v)$ of the 1000 random samples.

Table S3. *Simulation results for* $\widehat{\mathrm{QVE}}_\tau(v)$ *and* $\widehat{\mathrm{CQVE}}_\tau(v)$ *under model M4*

				$\widehat{\mathrm{QVE}}_\tau(v)$				$\widehat{\mathrm{CQVE}}_\tau(v)$			
τ	v	h	n	Bias	ESD	SD	CP	Bias	ESD	SD	CP
0.1	0.6	0.15	1000	0.029	0.408	0.379	0.922	0.014	0.131	0.124	0.944
			1500	0.021	0.333	0.317	0.927	0.017	0.109	0.110	0.929
		0.2	1000	0.016	0.347	0.325	0.933	0.007	0.122	0.115	0.940
			1500	-0.001	0.278	0.264	0.923	0.005	0.097	0.098	0.931
		h_{opt}	1000	0.006	0.297	0.280	0.928	0.001	0.111	0.104	0.942
			1500	0.008	0.261	0.232	0.952	0.007	0.094	0.088	0.956
	0.8	0.15	1000	0.014	0.296	0.269	0.934	0.018	0.153	0.141	0.949
			1500	0.029	0.243	0.230	0.935	0.021	0.126	0.126	0.937
		0.2	1000	0.008	0.255	0.232	0.944	0.010	0.145	0.134	0.946
			1500	0.006	0.206	0.199	0.918	0.005	0.116	0.115	0.941
		h_{opt}	1000	0.013	0.230	0.210	0.942	0.002	0.135	0.127	0.943
			1500	0.007	0.195	0.173	0.930	0.007	0.114	0.105	0.956
0.3	0.6	0.15	1000	0.019	0.335	0.322	0.928	0.022	0.118	0.110	0.950
			1500	0.016	0.268	0.250	0.948	0.019	0.110	0.108	0.949
		0.2	1000	0.009	0.282	0.273	0.932	0.012	0.109	0.107	0.940
			1500	0.010	0.232	0.216	0.943	0.011	0.100	0.101	0.935
		h_{opt}	1000	-0.001	0.241	0.232	0.935	0.007	0.102	0.098	0.940
			1500	-0.002	0.209	0.194	0.950	0.005	0.094	0.091	0.940
	0.8	0.15	1000	0.003	0.220	0.214	0.936	0.026	0.123	0.111	0.955
			1500	0.013	0.182	0.174	0.941	0.022	0.120	0.116	0.946
		0.2	1000	-0.001	0.188	0.182	0.942	0.016	0.107	0.112	0.952
			1500	0.007	0.154	0.149	0.939	0.013	0.111	0.109	0.943
		h_{opt}	1000	0.004	0.169	0.164	0.932	0.007	0.105	0.105	0.947
			1500	-0.002	0.142	0.141	0.942	0.004	0.095	0.099	0.956

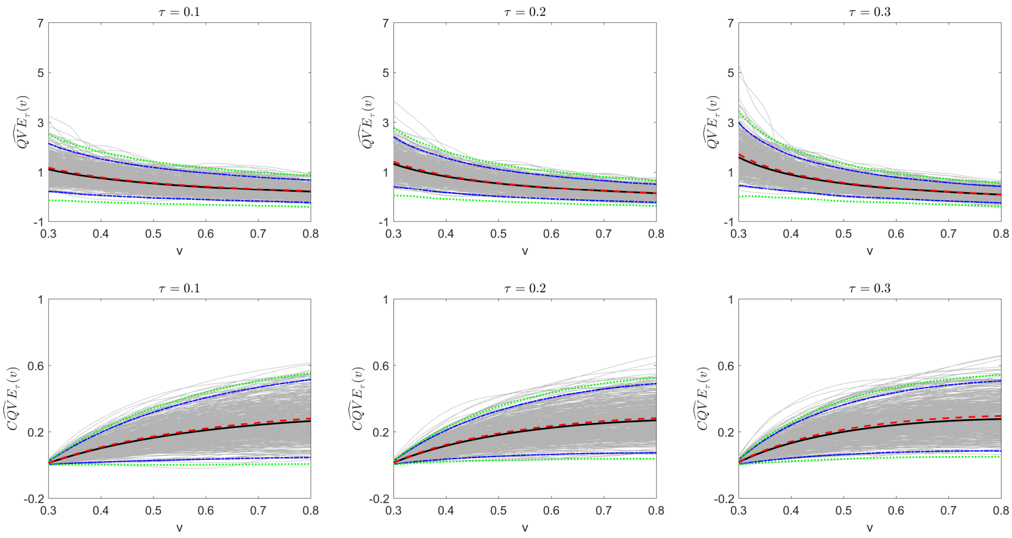

Fig. S4. Plots of the estimates for $\mathrm{QVE}_\tau(v)$ and $\mathrm{CQVE}_\tau(v)$ under model M3 with $n = 1000$. The bandwidth is chosen using the proposed cross-validation procedure. The sold lines are the true functions, the dashed lines are the averages of the estimates, the dash-dotted (blue) lines are the 95% pointwise confidence bands and the dotted (green) lines are the 95% simultaneous confidence bands. The gray lines are the corresponding estimates for $\mathrm{QVE}_\tau(v)$ and $\mathrm{CQVE}_\tau(v)$ of the 1000 random samples.

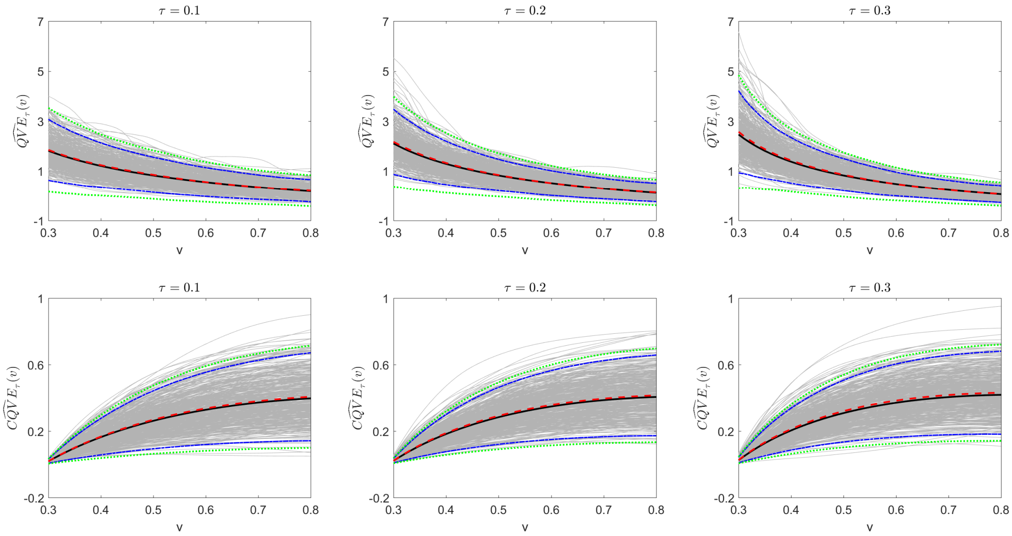

Fig. S5. Plots of the estimates for $\text{QVE}_\tau(v)$ and $\text{CQVE}_\tau(v)$ under model M4 with $n = 1000$. The bandwidth is chosen using the proposed cross-validation procedure. The sold lines are the true functions, the dashed lines are the averages of the estimates, the dash-dotted (blue) lines are the 95% pointwise confidence bands and the dotted (green) lines are the 95% simultaneous confidence bands. The gray lines are the corresponding estimates for $\text{QVE}_\tau(v)$ and $\text{CQVE}_\tau(v)$ of the 1000 random samples.

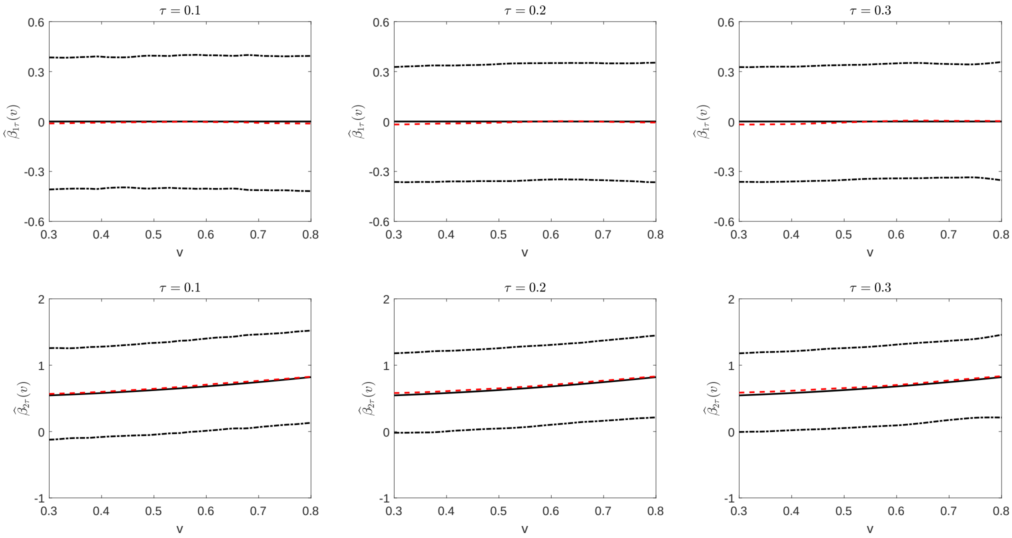

Fig. S6. Plots of the estimates for $\beta^*_{1\tau}(v)$ and $\beta^*_{2\tau}(v)$ under model M1 with $n = 1000$. The bandwidth is chosen using the proposed cross-validation procedure. The sold lines are the true functions, the dashed lines are the averages of the estimates, the dash-dotted lines are the 95% pointwise confidence bands.

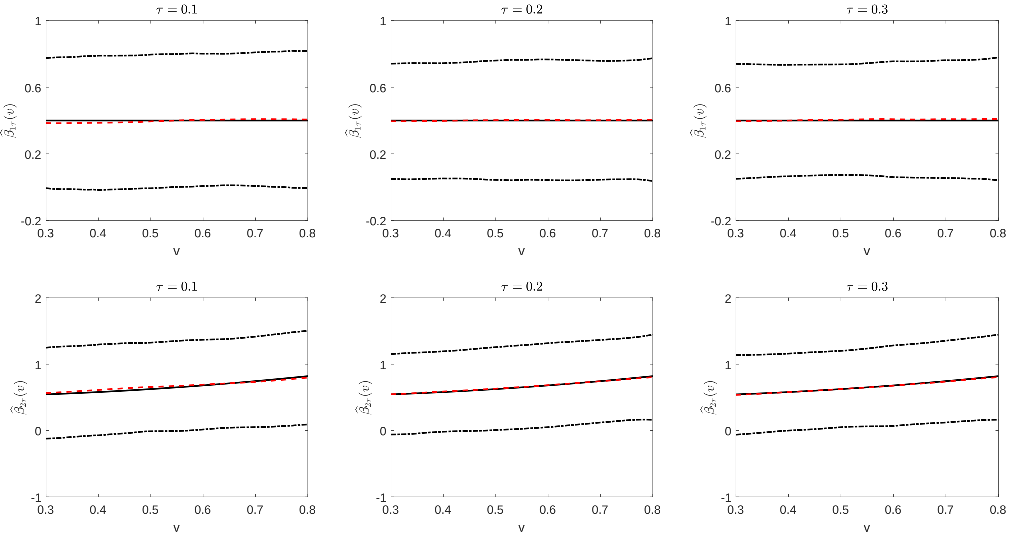

Fig. S7. Plots of the estimates for $\beta^*_{1\tau}(v)$ and $\beta^*_{2\tau}(v)$ under model M2 with $n = 1000$. The bandwidth is chosen using the proposed cross-validation procedure. The sold lines are the true functions, the dashed lines are the averages of the estimates, and the dash-dotted lines are the average of the 95% pointwise confidence bands in 1000 simulations.

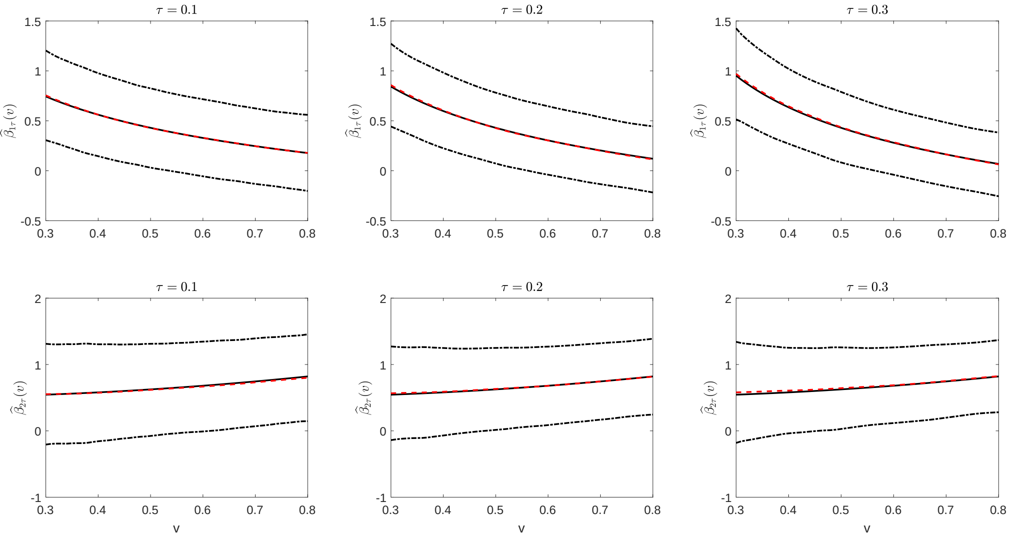

Fig. S8. Plots of the estimates for $\beta^*_{1\tau}(v)$ and $\beta^*_{2\tau}(v)$ under model M3 with $n = 1000$. The bandwidth is chosen using the proposed cross-validation procedure. The sold lines are the true functions, the dashed lines are the averages of the estimates, and the dash-dotted lines are the average of the 95% pointwise confidence bands in 1000 simulations.

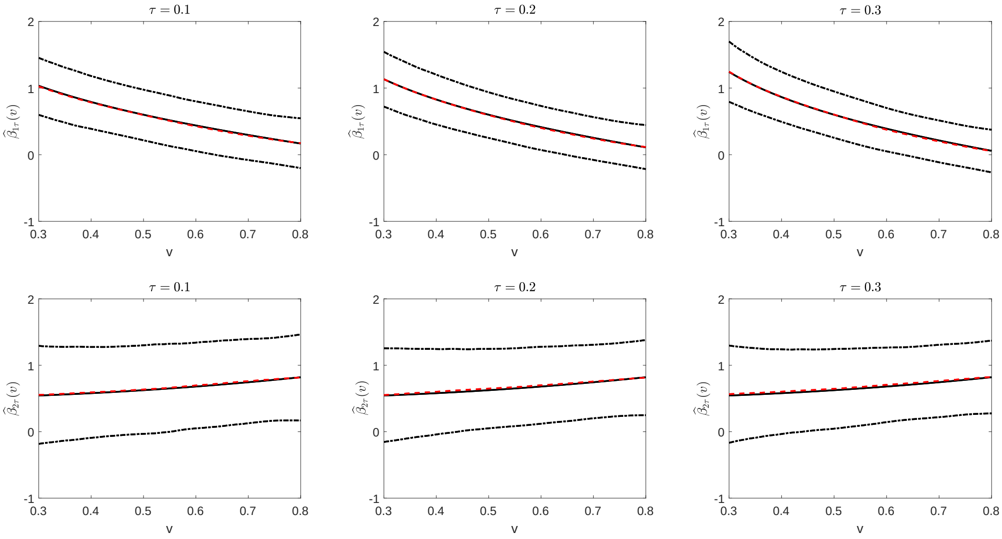

Fig. S9. Plots of the estimates for $\beta_{1\tau}^*(v)$ and $\beta_{2\tau}^*(v)$ under model M4 with $n = 1000$. The bandwidth is chosen using the proposed cross-validation procedure. The sold lines are the true functions, the dashed lines are the averages of the estimates, and the dash-dotted lines are the average of the 95% pointwise confidence bands in 1000 simulations.

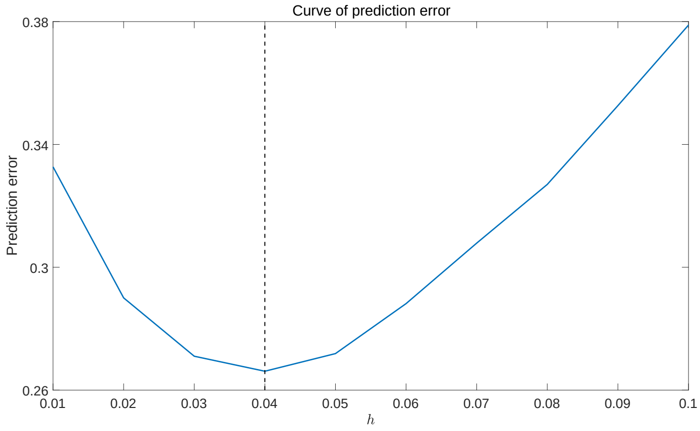

Fig. S10. Vaccine trial data analysis: Prediction errors versus bandwidths, indicating the optimal bandwidth $h_{\text{opt}} = 0.04$.

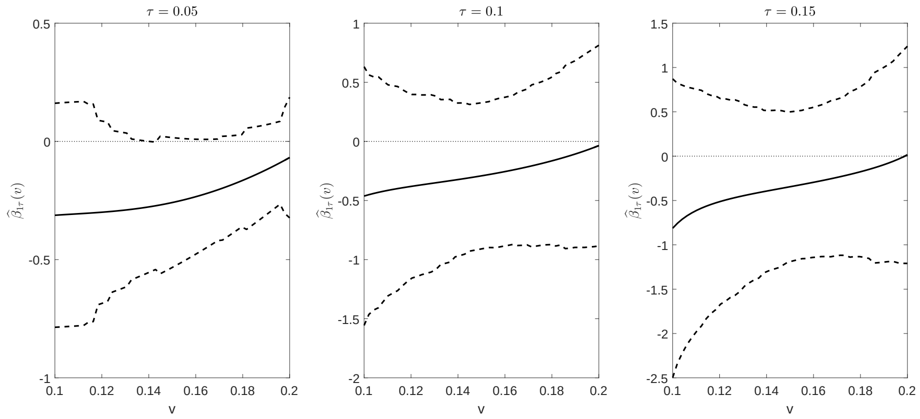

Fig. S11. Vaccine trial data analysis: The estimated curves of $\beta^*_{1\tau}(v)$ for treatment with $\tau = 0.05, 0.1$ and 0.15. The sold lines are the estimated functions and the dashed lines are the pointwise 95% confidence bands.

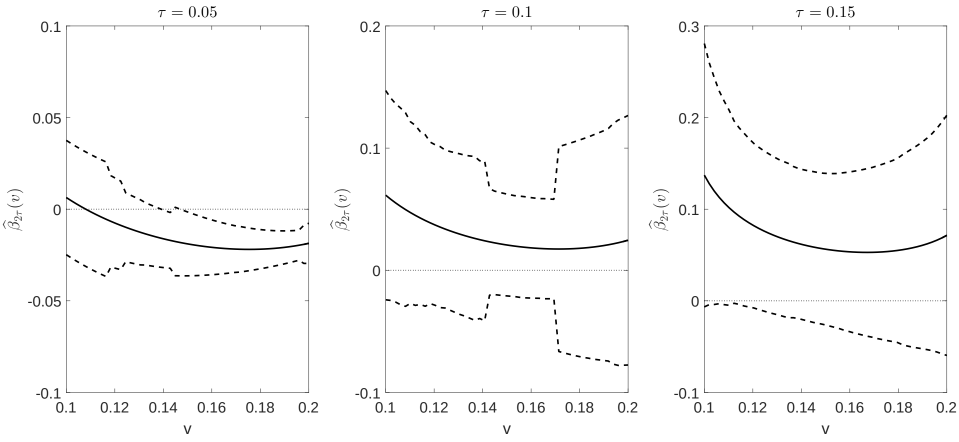

Fig. S12. Vaccine trial data analysis: The estimated curves of $\beta^*_{2\tau}(v)$ for age with $\tau = 0.05, 0.1$ and 0.15. The sold lines are the estimated functions and the dashed lines are the pointwise 95% confidence bands.

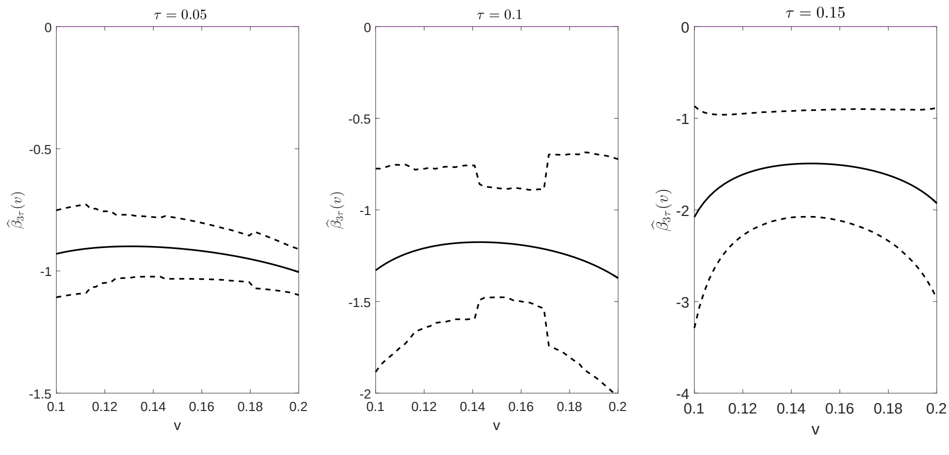

Fig. S13. Vaccine trial data analysis: The estimated curves of $\beta^*_{3\tau}(v)$ for behavior risk with $\tau = 0.05, 0.1$ and 0.15. The sold lines are the estimated functions and the dashed lines are the pointwise 95% confidence bands.

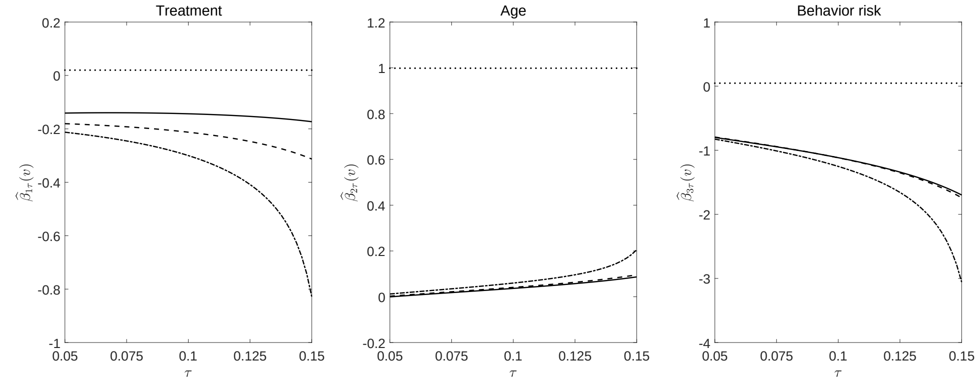

Fig. S14. Vaccine trial data analysis: The estimated curves of $\beta^*_{1\tau}(v)$, $\beta^*_{2\tau}(v)$ and $\beta^*_{3\tau}(v)$ for treatment, age and behavior risk. The dotted lines are the estimated curves using the method of Peng & Huang (2008), the dash-dotted, the dashed and the sold lines are the estimated curves using our method with $v = 0.1$, 0.15 and 0.2, respectively.
